# A combined gas-phase dissociative ionization, dissociative electron attachment and deposition study on the potential FEBID precursor [Au(CH_3_)_2_Cl]_2_

**DOI:** 10.3762/bjnano.14.98

**Published:** 2023-12-06

**Authors:** Elif Bilgilisoy, Ali Kamali, Thomas Xaver Gentner, Gerd Ballmann, Sjoerd Harder, Hans-Peter Steinrück, Hubertus Marbach, Oddur Ingólfsson

**Affiliations:** 1 Physikalische Chemie II, Friedrich-Alexander Universität Erlangen-Nürnberg, 91058 Erlangen, Germany; 2 Science Institute and Department of Chemistry, University of Iceland, Dunhagi 3, 107 Reykjavík, Iceland; 3 Inorganic and Organometallic Chemistry, Universität Erlangen-Nürnberg, 91058 Erlangen, Germany; 4 Carl Zeiss SMT GmbH, 64380 Roßdorf, Germany

**Keywords:** dissociative electron attachment, dissociative ionization, focused-electron-beam-induced deposition (FEBID), gold deposit, low-energy electrons, quantum chemical calculation, ultrahigh vacuum

## Abstract

Motivated by the potential of focused-electron-beam-induced deposition (FEBID) in the fabrication of functional gold nanostructures for application in plasmonic and detector technology, we conducted a comprehensive study on [Au(CH_3_)_2_Cl]_2_ as a potential precursor for such depositions. Fundamental electron-induced dissociation processes were studied under single collision conditions, and the composition and morphology of FEBID deposits fabricated in an ultrahigh-vacuum (UHV) chamber were explored on different surfaces and at varied beam currents. In the gas phase, dissociative ionization was found to lead to significant carbon loss from this precursor, and about 50% of the chlorine was on average removed per dissociative ionization incident. On the other hand, in dissociative electron attachment, no chlorine was removed from the parent molecule. Contrary to these observations, FEBID in the UHV setup was found to yield a quantitative loss and desorption of the chlorine from the deposits, an effect that we attribute to electron-induced secondary and tertiary reactions in the deposition process. We find this precursor to be stable at ambient conditions and to have sufficient vapor pressure to be suitable for use in HV instruments. More importantly, in the UHV setup, FEBID with [Au(CH_3_)_2_Cl]_2_ yielded deposits with high gold content, ranging from 45 to 61 atom % depending on the beam current and on the cleanliness of the substrates surface.

## Introduction

In recent years, gold nanostructures have received much attention owing to their dielectric properties [1], their biocompatibility [2], and their electrical properties [3–4], which enable a multitude of exciting applications in the field of nanotechnology. These include, but are not limited to electronic interconnects [5], metamaterials [6], growth substrates for nanowires and nanotubes [7], and complex plasmonic structures [8–9]. For the latter application, mastery over the shape as well as accurate control of the distribution of the nanostructures are critical for the enhancement of absorption and controlled scattering of light [10]. Focused-electron-beam-induced deposition (FEBID) is a direct writing method for controlled deposition/fabrication of nanostructures on either flat or nonflat surfaces. It offers excellent shape control and thus the potential to widen the scope of applicable nanomaterials. In FEBID, a focused electron beam is directed onto the surface of a substrate in close proximity to a gas inlet, through which a precursor compound is supplied to deliver the material for the nanostructures to be built. For metallic structures, these precursor molecules are commonly organometallics that adsorb on the substrate and are decomposed by the electron beam irradiation. Ideally, a pure metal is deposited while fragmented volatile ligands are pumped away [11–13].

Several parameters affect the FEBID process, including the electron beam energy and current, the substrate material, the environment inside the deposition chamber, and the composition of the precursor [14–17]. Heretofore, various chemical vapor deposition (CVD) precursors have been applied for FEBID depositions. For gold nanostructures, these include, for example, dimethyl(acetylacetonate)gold(III) (Au(acac)(CH_3_)_2_), dimethyl(trifluoroacetylacetonate)gold(III) (Au(tfac)(CH_3_)_2_), and dimethyl(hexafluoroacetylacetonate)gold(III) (Au(hfac)(CH_3_)_2_) [18]. Although these precursors have proven suitable for CVD, in FEBID their application has mainly resulted in amorphous matrixes of carbon with embedded metal crystallites and a gold content of 2–3 atom % [19], 10–40 atom % [20], and 8–20 atom % [21], respectively. This is most likely due to the fact that the CVD process is thermally driven, while in FEBID, the precursor fragmentation is primarily electron driven. This may partly explain the insufficient metal content achieved when using CVD precursors in FEBID [18]. In this context, efforts have been made to design gold precursors optimized for FEBID. Arguably, the most noticeable of these are chloro(carbonyl)gold(I) (Au^I^Cl(CO)) [22] and chloro(trifluorophosphine)gold(I) (Au^I^Cl(PF_3_)) [23]. These precursors have enabled depositions of ≈95 atom % Au and a resistivity of Au grains as low as 22 µΩ, respectively. However, the short lifetime of both precursors, which results from their moisture sensitivity and thermal instability, has limited their applicability.

In FEBID, the irradiation of the substrate with a high-energy focused electron beam results in elastic and inelastic electron scattering, including ionizing events. The latter leads to the production of numerous reactive, low-energy scattered and secondary electrons. These play a significant role in the precursor decomposition and thus in the deposit formation [16]. Hence, the decomposition of the precursor molecules is not only effectuated by the primary electron beam. In fact, the reactivity of these low-energy electrons [24] may even determine the fragmentation of the precursor molecules, which in turn is critical to the resulting purity of the FEBID deposits. In general, electron-induced fragmentation processes are categorized as dissociative ionization (DI), dissociative electron attachment (DEA), dipolar dissociation (DD), and neutral dissociation (ND) [25]. To fully comprehend the electron–molecule interactions in FEBID, it is critical to understand the extent and nature of these processes and how they are reflected in the deposit formation from individual precursors or specific ligand structures. A very interesting approach in this direction was recently introduced by Jurczyk et al. [26] under the term focused-electron-beam-induced mass spectrometry (FEBiMS). In this approach, ion-extraction mass spectrometry, in close proximity to the forming FEBID structure, is used to analyze the charged, desorbing ligand fragments. Another approach in this direction is to combine ultrahigh-vacuum (UHV) surface science studies and mass spectrometry in high-vacuum (HV) gas-phase investigations [27–28]. In this context, surface science experiments allow for electron-dose-dependent studies of the elemental composition of the deposit, and desorbing ligands may be monitored by means of mass spectrometry. On the other hand, gas-phase studies using controllable, quasi-monoenergetic electron beams under single collision conditions, provide information on the electron energy dependence and extent of the individual fragmentation processes [28]. A number of such comparative gas-phase and surface science studies have been carried out in the past using a 500 eV flood gun in the surface studies [29–30], and also in combination with higher energy FEBID studies [30–31]. In a recent study [32], we took a similar approach and investigated (CH_3_)AuP(CH_3_)_3_ as a potential gold precursor for FEBID. We used gas-phase experiments under single-collision conditions and quantum mechanical calculations for data interpretation, in combination with FEBID in an UHV setup. The results of this study demonstrated that at 5 keV electron energy, FEBID deposits with 31–34 atom % Au content were attainable with this precursor in the UHV setup. A close-to-complete phosphorous removal was observed and the Au/C ratio of the deposit was found to be 1:2. This corresponds to the average carbon loss per incident beam found in the DI gas-phase experiment, and was consistent with the dominating reaction pathways as determined by the quantum chemical calculations. However, in one specific channel in the DI gas-phase study, a significant retention of the phosphorous at the gold precursor was found indicating significant surface effects.

In the current study, we extended this approach to investigate the deposition of Au using [Au(CH_3_)_2_Cl]_2_ as a potential FEBID precursor. The FEBID in an UHV setup was conducted, in conjunction with a gas-phase study on the electron energy dependence of the fragmentation of this compound through DI and DEA. Moreover, quantum chemical calculations were used to determine the dominating reaction pathways. The UHV FEBID results are discussed in the context of the observed DI and DEA fragmentation processes, and also in the context of a previous FEBID study of this precursor under HV, conducted by van Dorp et al. [33] In that study, a promising Au content of 29–41 atom % was achieved without additional substrate purifications. In the current study, we found the Au content to be further improved to reach about 50 atom % in the UHV setup without pretreatment of the substrate surface. With a pretreated surface, a gold content of 61 atom % was reached.

## Results and Discussion

### FEBID on SiO_2_ (500 nm)/Si(111)

**Figure 1:**
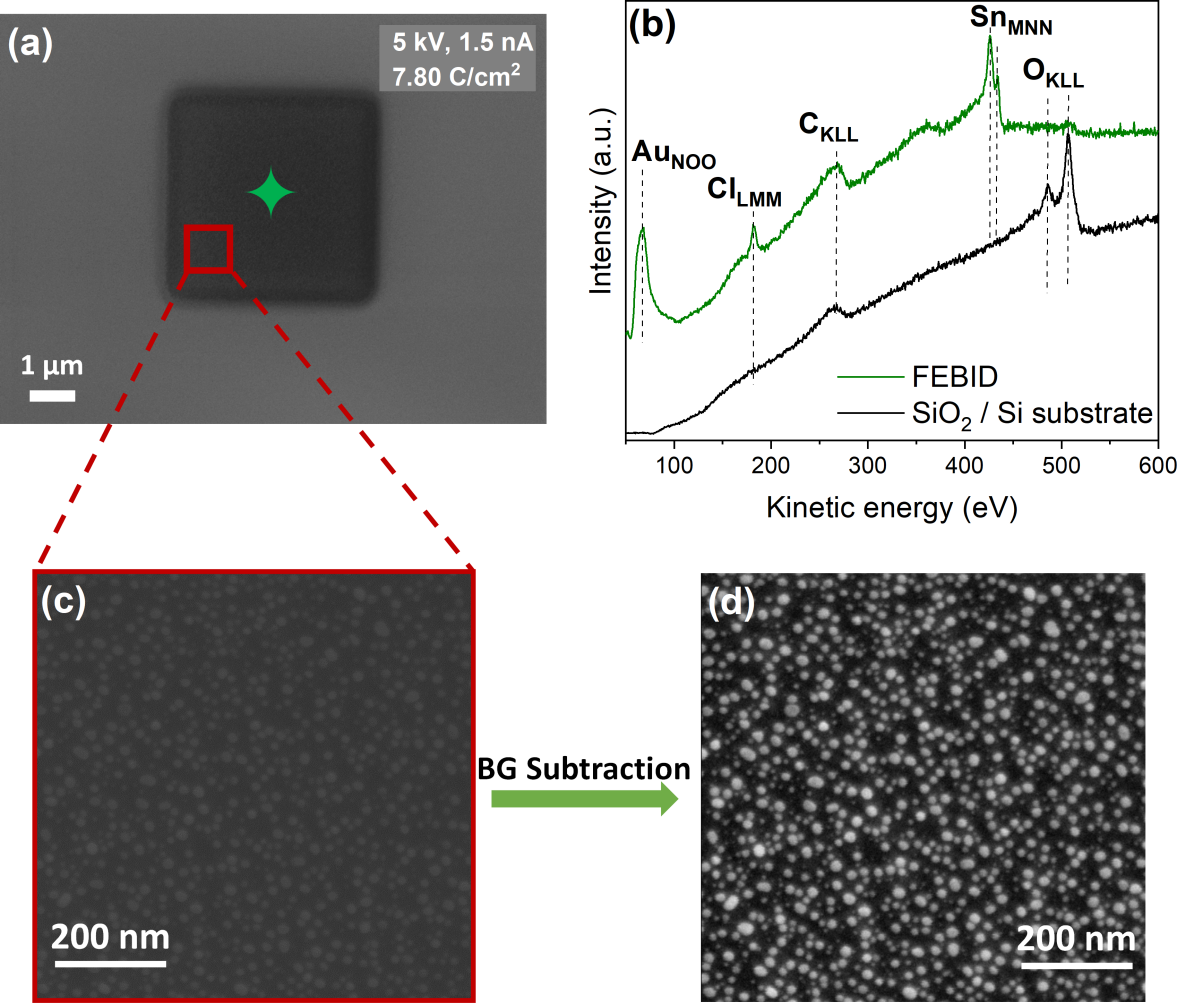
(a) An SEM image of a 4 × 4 µm^2^ FEBID structure deposited on SiO_2_ from [Au(CH_3_)_2_Cl]_2_ with an electron dose of 7.80 C/cm^2^ using the electron beam parameters of 5 keV and 1.5 nA. (b) An AES plot of the SiO_2_ substrate prior to deposition (black line) and from the FEBID structure (green line); the green-colored star in (a) indicates the position where the spectrum was acquired. (c) Magnified image from the area within the red-colored square shown in (a). (d) The same image as shown in (c) after the background subtraction process was applied using the ImageJ program [35].

In this experiment, 4 × 4 µm^2^ FEBID structures were written with [Au(CH_3_)_2_Cl]_2_ as the precursor using an acceleration voltage of 5 keV and a beam current of 1.5 nA. The fabricated structures were examined with scanning electron microscopy (SEM) and Auger electron spectroscopy (AES). Figure 1a depicts an SEM image of the FEBID deposit created with an electron exposure of 7.80 C/cm^2^. The position of the corresponding AES analysis is marked in Figure 1a by a green-colored star. The AES spectra acquired on the bare substrate and the deposit are shown in Figure 1b. On the pristine SiO_2_ (500 nm)/Si(111) substrate (black spectrum), only two main AES signals are visible: The peak at 272 eV is attributed to C_KLL_ Auger transitions of carbon [34], and the peaks at 468, 483, and 503 eV to O_KLL_ Auger transitions of SiO_2_ [34].

After deposition, AES signals at 69, 181, 272, and 430 eV are present. These are assigned to the Au_NOO_, Cl_LMM_, C_KLL_, and Sn_MNN_ Auger transitions [34], respectively (Figure 1b, green spectrum). The broad and small peak at approximately 367 eV is attributed to an Sn signal [34]. The contamination with Sn is from the synthesis process of the [Au(CH_3_)_2_Cl]_2_ precursor, which involves SnMe_4_ as a methylation agent [36]. The elemental composition of the FEBID structure was calculated using the relative sensitivity factors (S) [37], that is, S_Au_: 0.21; S_Cl_: 0.69; S_C_: 0.08; S_Sn_: 0.53. From these, the atomic concentration of the deposit was found to be 51 atom % Au, 2 atom % Cl, 42 atom % C, and 5 atom % Sn. Considering the ratio of the Sn_MNN_ signal to that of the remaining O_KLL_ signal from the deposit, compared to that expected for stannic oxide [38] SnO_2_, it is clear that the Sn impurity is predominantly elemental rather than on the oxidized form. A magnification of a selected area of the SEM image shown in Figure 1a is depicted in Figure 1c, where nanoparticles in the deposition are noticeable, although the picture is somewhat blurry. To better visualize the observed nanoparticles, a background subtraction was performed with the image enhancement program ImageJ [35]. The image after the background subtraction is shown in Figure 1d, where the particles can be more clearly distinguished. After background subtraction, some of the deposited nanoparticles appear facetted; however, the majority are spherical.

### HAADF-STEM on FEBID (SiO_2_ (500 nm)/Si(111))

**Figure 2:**
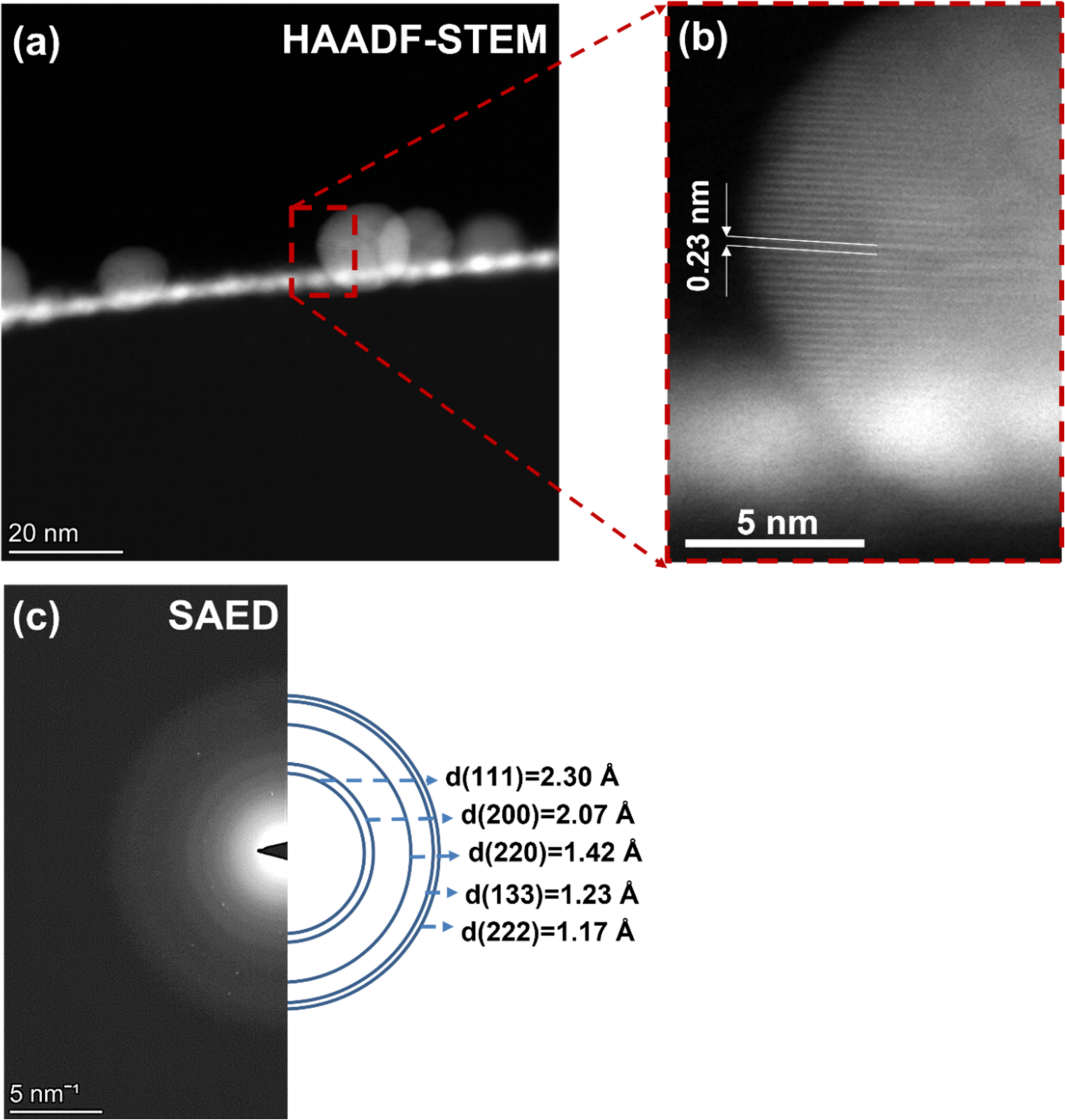
(a) A HAADF-STEM image of a FEBID gold nanoparticle. (b) Enlarged image of the area depicted with red-dashed lines in (a), showing the interplanar distance of 0.23 nm between the {111} planes of the FCC lattice. (c) A SAED pattern of the FEBID gold nanoparticles, compared with the lattice spacings (d-spacings) of FCC gold.

As a next step, high-angle annular dark-field scanning transmission electron microscopy (HAADF-STEM) was performed to analyze the morphology of the deposited nanoparticles. For this purpose, several FEBID structures were prepared on the SiO_2_ substrate with the size of 4 × 4 µm^2^ and an electron dose of 7.80 C/cm^2^. For the HAADF-STEM measurements, a lamella was prepared with a thickness of approx. 100 nm and a width of approx. 4 µm (Supporting Information File 1, Figure S1). In Figure 2a, the HAADF-STEM image of deposited nanoparticles is shown, revealing a nearly uniform spatial distribution of nanoparticles with a size lower than 5 nm. Nonuniformly distributed nanoparticles with larger sizes (≈15–20 nm) were also observed. The magnified image of a selected larger nanoparticle from Figure 2a is shown in Figure 2b.

The determined fringe spacing of that particle is ≈0.23 nm, which is consistent with the spacing between the (111) planes of a face-centered cubic (FCC) gold nanoparticle [39–40]. The crystallinity of the gold nanoparticles was also investigated by using selected area electron diffraction (SAED) pattern shown in Figure 2c. The relatively bright circular patterns indicate polycrystallinity of the deposits. For comparison, the lattice spacings (d-spacings) of 2.30, 2.07, 1.42, 1.23, and 1.17 Å [41], corresponding to the (111), (200), (220), (133), and (222) growth planes, respectively, of the FCC lattice of crystalline gold is also shown in Figure 2d.

### FEBID on SiO_2_ (500 nm)/Si(111) at different beam currents

**Figure 3:**
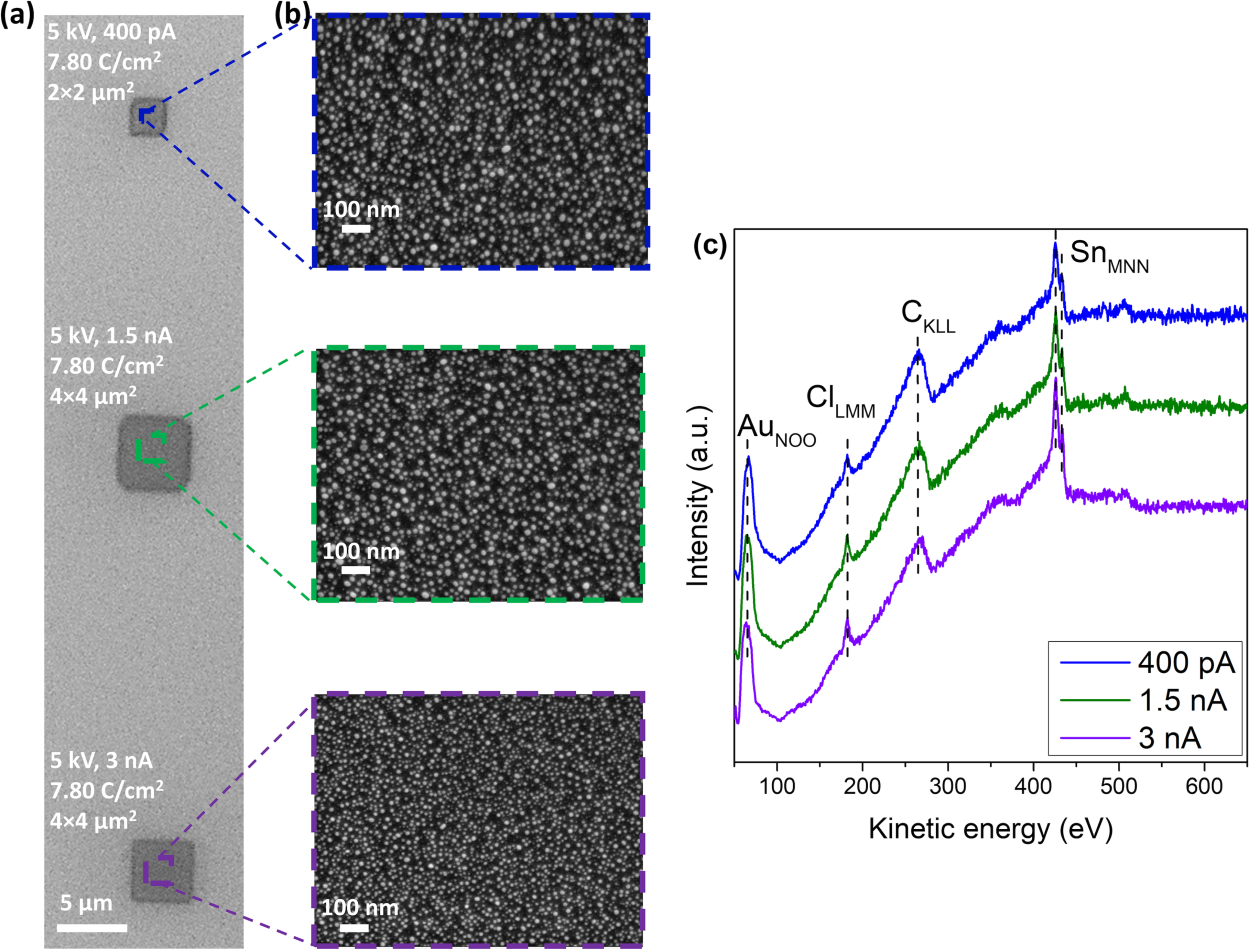
(a) An SEM image of FEBID structures deposited on SiO_2_ from [Au(CH_3_)_2_Cl]_2_ with an electron dose of 7.80 C/cm^2^ using a 5 keV electron beam and different beam currents of 0.4 nA, 1.5 nA, and 3 nA. (b) Magnified images of FEBID structures from (a). (c) An AES plot of the FEBID structures deposited with 0.4 pA, 1.5 nA, and 3 nA depicted with blue, green, and purple lines, respectively.

The FEBID deposits were also prepared with [Au(CH_3_)_2_Cl]_2_ using beam currents of 0.4 nA (deposit size: 2 × 2 μm^2^), 1.5 nA (deposit size: 4 × 4 μm^2^), and 3 nA (deposit size: 4 × 4 μm^2^). The other deposition parameters (electron dose: 7.80 C/cm^2^ and acceleration voltage: 5 keV) were the same in all three experiments. The FEBID structures were investigated by SEM and noncontact atomic force microscopy (AFM). Figure 3a shows the SEM images of the deposits along with the respective deposition parameters. Magnified sections from these SEM images are shown in Figure 3b. Auger electron spectroscopy was performed on these structures to determine their composition and to better understand the effect of different beam currents on the compositions. The respective spectra are shown in Figure 3c.

A careful investigation of the SEM images reveals that the particle size gets smaller when the current is increased, as is clearly discernible from Figure 3b when comparing deposition at a beam current of 1.5 and 3 nA. For detailed particle analysis, the ImageJ software [35] was used to obtain the numbers of nanoparticles and their mean diameter. As mentioned before, the observed gold nanoparticles (Figure 1d) have irregular shapes. Therefore, the mean Feret diameter, which gives the average value over all possible orientations was used (see Supporting Information File 1, Figure S2). The average particle sizes determined from the SEM images were found to be similar at beam currents of 0.4 and 1.5 nA (i.e., 9.8 and 10.1 nm, respectively). At 3 nA, on the other hand, a clear size reduction to 8.2 nm is observed in the SEM images. This size reduction with increasing deposition current is even clearer from the AFM images as discussed in the following section. From the AES data shown in Figure 3c, the atomic concentrations of the structures were calculated: At 0.4 nA, the composition was found to be 45 atom % Au, 1 atom % Cl, 49 atom % C, 5 atom % Sn; at 1.5 nA it was found to be 50 atom % Au, 2 atom % Cl, 42 atom % C, 6 atom % Sn; and at 3 nA, it was found to be 52 atom % Au, 2 atom % Cl, 38 atom % C, 8 atom % Sn. For ease of comparison, the respective elemental compositions are also reported in Table 1 along with the composition of a deposit on thermally cleaned Si(111) at a 1.5 nA beam current, as discussed in the next section.

**Table 1:** Elemental composition (atom %) of depositions on SiO_2_ (500 nm)/Si(111) at different beam currents (nA). Also shown is the elemental composition of a deposition on thermally cleaned Si(111).

SiO_2_ (500 nm)/Si(111)				

Current	Au	C	Cl	Sn

0.4	45	49	1	5
1.5	50	42	2	6
3.0	52	38	2	8

Thermally cleaned Si(111)				

Current	Au	C	Cl	Sn

1.5	61	35	1	3

Clearly, the increase in gold content with increasing deposition current is correlated with the decrease in carbon content, which is also reflected in the proportionally increasing Sn contaminations showing the same trend as that of gold. This is more evident from the reduction of the carbon peak areas in the AES, which is ≈36% when comparing the depositions at 0.4 and 3 nA, and ≈14% when comparing the depositions at 0.4 and 1.5 nA. We thus attribute the observed size reduction of the deposited gold particles with increasing deposition current to the decrease in carbon content. A similar size reduction of gold nanoparticles has been reported for post-deposition oxidative purification of FEBID deposits, where carbon removal led to ≈18% height reduction of the respective nanoparticles [42]. Notwithstanding, changes in the deposition time and in the associated different volume of the deposited material may also contribute to the observed particle size reduction.

### AFM of FEBID on (SiO_2_ (500 nm)/Si(111)) at different beam currents

**Figure 4:**
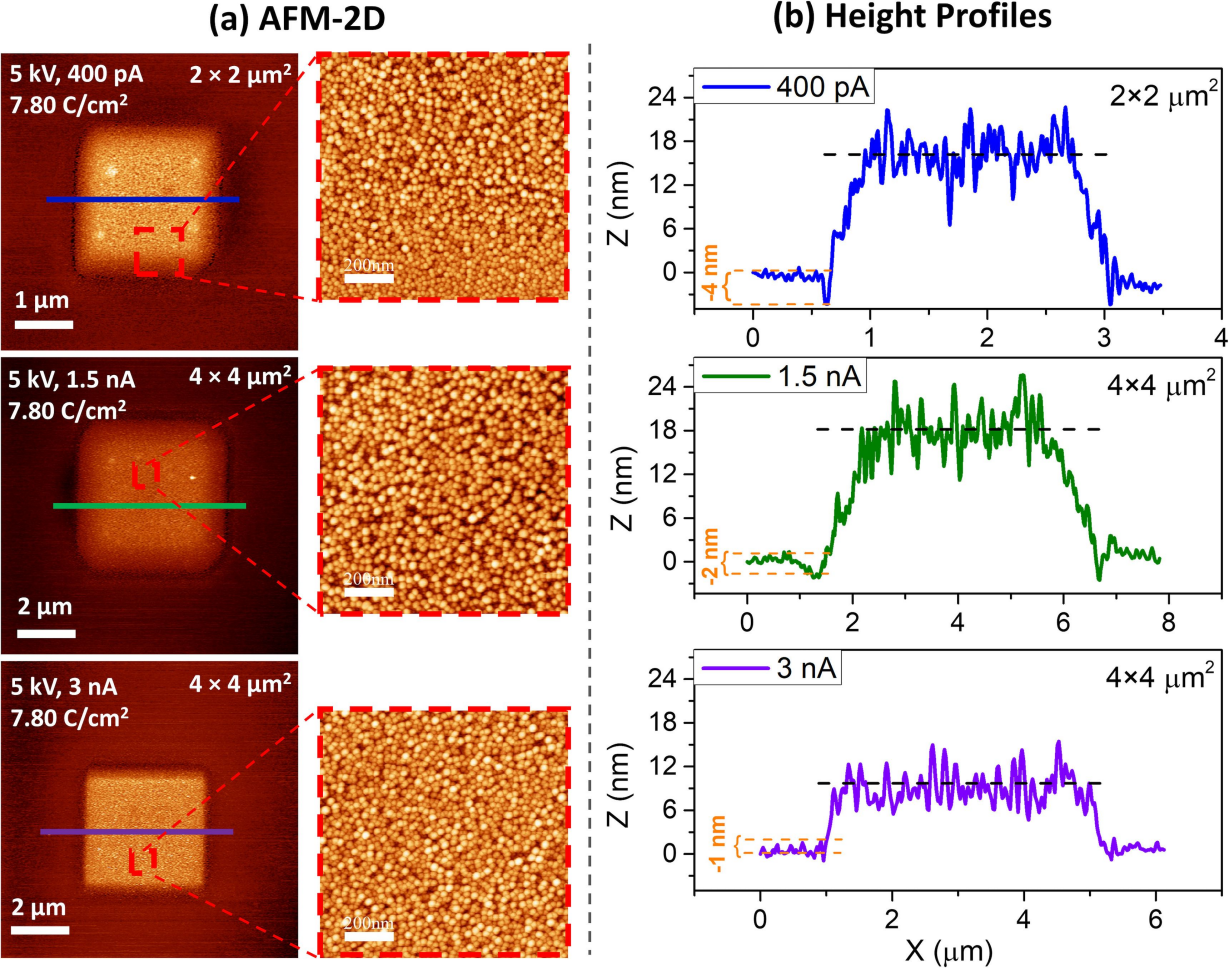
(a) A set of 2D AFM images and magnified AFM images (red-dashed squares). (b) The corresponding line profiles for the FEBID structures produced with an electron dose of 7.80 C/cm^2^ using the beam currents of 0.4 nA (blue line), 1.5 nA (green line), and 3 nA (purple line).

In order to obtain complementary information on the structures deposited with different beam currents, noncontact AFM was used to investigate the height of the deposits and their particle size. Figure 4a and Figure 4b depict the 2D AFM images and magnified sections of these, respectively. The corresponding height profiles are shown in Figure 4c. The magnified sections of the 2D AFM images (Figure 4b) show the same trend as observed in the SEM images shown in Figure 3b. The size of the gold nanoparticles is approximately the same for the FEBID structures written with 0.4 and 1.5 nA, while they are smaller in the deposit written with 3 nA beam current. The average particle sizes obtained from the AFM images are approx. 10.4 nm for 0.4 nA, 9.5 nm for 1.5 nA, and 7.0 nm for 3 nA (Supporting Information File 1, Figure S3b). These values are in good agreement with the values obtained from the SEM images (9.8 nm – 0.4 nA; 10.1 nm – 1.5 nA; 8.2 nm – 3 nA). Notably, the line profiles in Figure 4b for the structures created with 0.4 and 1.5 nA reveal thicknesses of the deposits of ≈17 nm, while the thickness of the deposit written with 3 nA is only ≈9 nm. As aforementioned, we attribute the size reduction, at least in part, to a more efficient carbon removal at higher currents. The same applies to the observed reduction in thickness with increasing beam current. However, the reduction of volume growth rate per dose at higher currents and the thickness of the deposits may also affect the observed particle size.

Interestingly, the height profiles of depositions also change according to the applied beam current (Figure 4b). For example (most significant in the height profile of the 0.4 nA deposition, Figure 4b, blue line), there is a negative dip at the edge of the deposits, indicated by the orange dashed lines. It is important to note that this negative dip is also observed for other line profiles throughout the deposit. Therefore, the negative dips are present in the entire structure (Supporting Information File 1, Figure S4). This negative dip can also be seen for the depositions created with 1.5 and 3 nA beam currents, depicted in Figure 4b with green and purple lines, respectively. However, the depth of the dip decreases with increasing applied beam current. This indicates that an etching process occurs simultaneously with the deposition process, wherein the etching effect is less pronounced than that of the deposition for all beam currents. Similar etching effects were observed with other halogenated precursors, where it was reported that one of the expected effects when working with halogen-based precursors is the observation of etching as well as deposition [22,43]. In these studies, the release of halogen ligands was indicated as the main reason for the etching process.

### FEBID on thermally cleaned Si(111)

**Figure 5:**
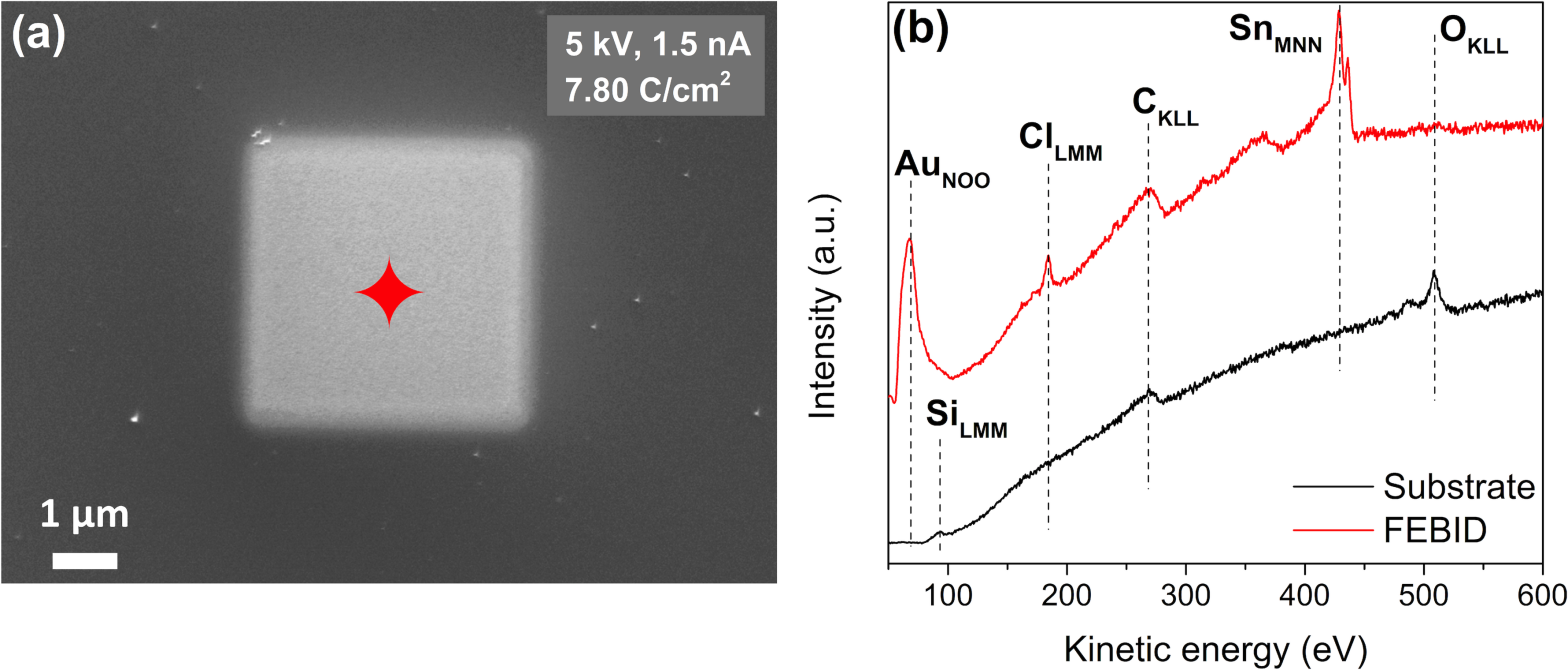
(a) An SEM image of a 4 × 4 µm^2^ FEBID structure deposited on Si(111) from [Au(CH_3_)_2_Cl]_2_ with an electron dose of 7.80 C/cm^2^ using the electron beam parameters of 5 keV and 1.5 nA. (b) An AES plot of the Si(111) substrate prior to deposition (black line) and the result from the FEBID structure (red line). The red-colored star in (a) indicates the position where the spectrum was acquired.

In several UHV-FEBID studies [43–45] it has been shown that an UHV setup alone is not sufficient to produce FEBID structures with relatively low organic contaminations. In addition, a comparably clean and well-defined substrate also helps to increase the metal content. Thus, the Si(111) substrate was sputtered using Ar^+^ for 45 minutes (*V*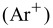 = 1 eV, *P*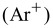 = 4 × 10^−6^ mbar) and subsequently annealed up to 823 K under an oxygen atmosphere for 90 minutes to demonstrate the effect of surface preparation (reduction of C and O contaminants) on the quality of deposition. After preparation, AES was performed to check the surface cleanliness and to compare with the uncleaned surface (Supporting Information File 1, Figure S5). The Supporting Information File 1, Figure S5, clearly shows that the carbon (C_KLL_ at 272 eV) and oxygen peaks (O_KLL_ at 508 eV) were reduced (by ≈17% for C, ≈67% for O), and thus the Si_LMM_ peak at 92 eV became observable. Using the cleaned sample, a FEBID experiment with the [Au(CH_3_)_2_Cl]_2_ precursor was performed to create 4 × 4 µm^2^ structures using the same parameters as were used for the deposits depicted in Figure 1. The results were analyzed with SEM and AES, see Figure 5. The red-colored star indicates the AES measured point.

The AES plot (red-colored line) shows the following peaks: Au_NOO_ at 69 eV, Cl_LMM_ at 181 eV, C_KLL_ at 272 eV, and Sn_MNN_ at 430 eV [34], yielding atomic concentrations of 61 atom % Au, 1 atom % Cl, 35 atom % C, and 3 atom % Sn, respectively. A comparison to Figure 1b reveals an increase of Au content by 10%, while the C content decreases by 10%. In a previous study, the same precursor (i.e., [Au(CH_3_)_2_Cl]_2_) was used to create FEBID deposits on a SiO_2_ substrate by using 5 keV and 0.1/0.4 nA in an HV atmosphere [33]. The composition of the structures was checked via energy dispersion X-ray (EDX) mapping and reported to be 29–41 atom % Au, 2–6 atom % Cl, and 53–68 atom % C. The SEM images of the FEBID deposits also revealed grainy structures. These concentrations, reported in the reference study, support the idea of complete Cl ligand desorption and incorporation of both CH_3_ ligands into the deposit. The main difference between the work at hand and the aforementioned study in HV, is that this study was carried out in UHV with a higher electron beam current of 1.5 nA. However, it should be mentioned that the AES used here is surface sensitive as compared to EDX, which is bulk sensitive. As the synthesis and purification routes in both HV and the current UHV studies are apparently the same, it is surprising that no Sn impurities were reported in the deposits made under HV. As the information on the synthesis route is limited in the HV study reported, we can only speculate that a different methylation agent may have been used (i.e., one that did not contain tin). An alternative explanation may lie in the usage of different characterization tools (i.e., EDX and AES). From both the UHV and HV FEBID results, one can conclude that the Cl ligands are completely removed and desorbed under the impact of the electron beam. The ease of Cl ligand desorption during electron beam deposition has also been addressed in several previous studies [22–23]. Notably, the UHV-FEBID results yield 10–20 atom % higher Au content than those reported in the HV study. Therefore, the reaction pathway of [Au(CH_3_)_2_Cl]_2_ can be suggested as:




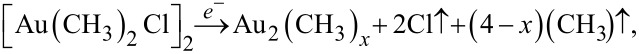




where Au_2_(CH_3_)*_x_* is the deposited material, while 2Cl and (4 − *x*)(CH_3_) are desorbed from each molecule. We note that these may be desorbed as Cl_2_, CH_3_Cl, or CH_3_CH_3_ as discussed in the next section. Further, we expect the final deposit to rather result from electron-induced secondary and tertiary reaction than from a single electron precursor interaction. According to the AES depicted in Figure 1b and Figure 5b, *x* can be inferred to be 1–2.

### Gas-phase studies

**Figure 6:**
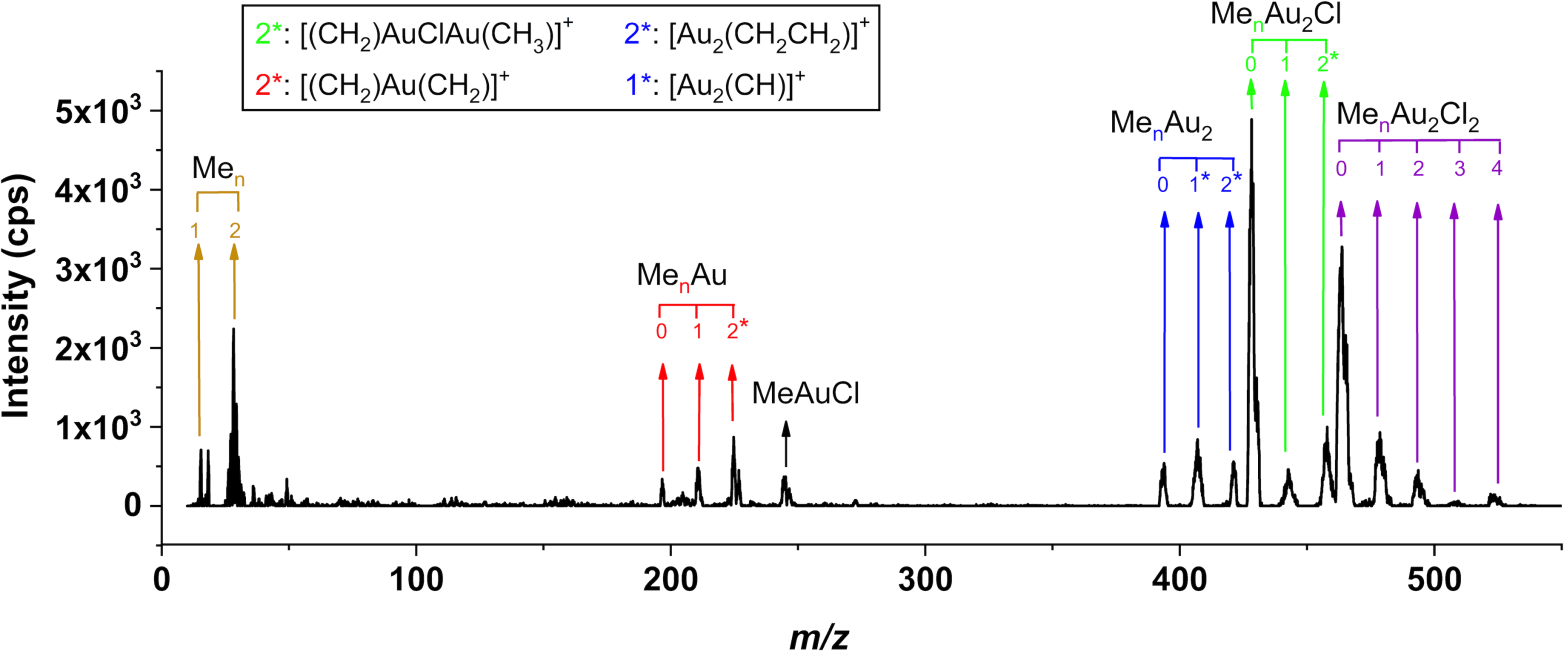
Positive ion mass spectrum of electron impact ionization and dissociation of [Au(CH_3_)_2_Cl]_2_ recorded at an incident electron energy of 50 eV.

Figure 6a shows a positive ion mass spectrum of [Au(CH_3_)_2_Cl]_2_, recorded for the *m*/*z* range from 10 to 550 at a 50 eV electron impact energy. A rich fragmentation pattern, characterized by progressive loss of methyl groups, is observed.

The first progression is that of the molecular cation at *m*/*z* 524 followed by a sequential loss of methyl ligands, appearing at *m*/*z* 509, 494, 479, and 464, with the most significant contribution being from the loss of all four methyl ligands at *m*/*z* 464. The second progression shows the loss of one chlorine atom and two, three, and four methyl ligands at *m*/*z* 458, 444, and 429, respectively. From these, *m*/*z* 458 has lost an additional hydrogen and *m*/*z* 444 overlaps with lesser contributions from *m*/*z* 443, which we also attribute to additional hydrogen loss. Similar to the preceding progression, the loss of all four methyl ligands, *m*/*z* 429, is also the dominating contribution here. The third progression shows the loss of both chlorines in combination with the sequential loss of two, three, and four methyl ligands at *m*/*z* 422, 408, and 394, respectively. Here, the contributions are of similar intensity, although the loss of three methyl ligands, *m*/*z* 408, is slightly more apparent. Lesser contribution is also observed at *m*/*z* 407 and is attributed to additional hydrogen loss as compared to that of *m*/*z* 408. The last progression is from the loss of both chlorine atoms along with one gold atom, and two and three methyl groups and is observed at *m*/*z* 227 and 225, 212 and 197, respectively. From these, *m*/*z* 225 is ascribed to the loss of two methyl groups and two additional hydrogens, and 197 represents Au^+^ (i.e., the elemental gold). Additionally, *m*/*z* 247 is observed with fair intensity and we attribute this fragment to the loss of three methyl ligands in combination with the loss of one chlorine and one gold atom (i.e., [Au(CH_3_)Cl]^+^). There are some broad low-intensity impurity contributions in the EI MS in the *m*/*z* range of SnCl (150–160) and SnCl(CH_3_) (200–210). However, these are low intensity and cannot be unambiguously assigned from their isotope distribution. The most significant low *m*/*z* contributions are around *m*/*z* 28 and 15. The contributions at and around *m*/*z* 28 are predominantly from the background gas in the chamber, including N_2_, but may also contain C_2_H*_n_* contributions from rearrangement reactions of [Au(CH_3_)_2_Cl]_2_ upon ionization. Similarly, *m*/*z* 15 is in part attributed to CH_3_^+^ resulting from DI of [Au(CH_3_)_2_Cl]_2_.

**Figure 7:**
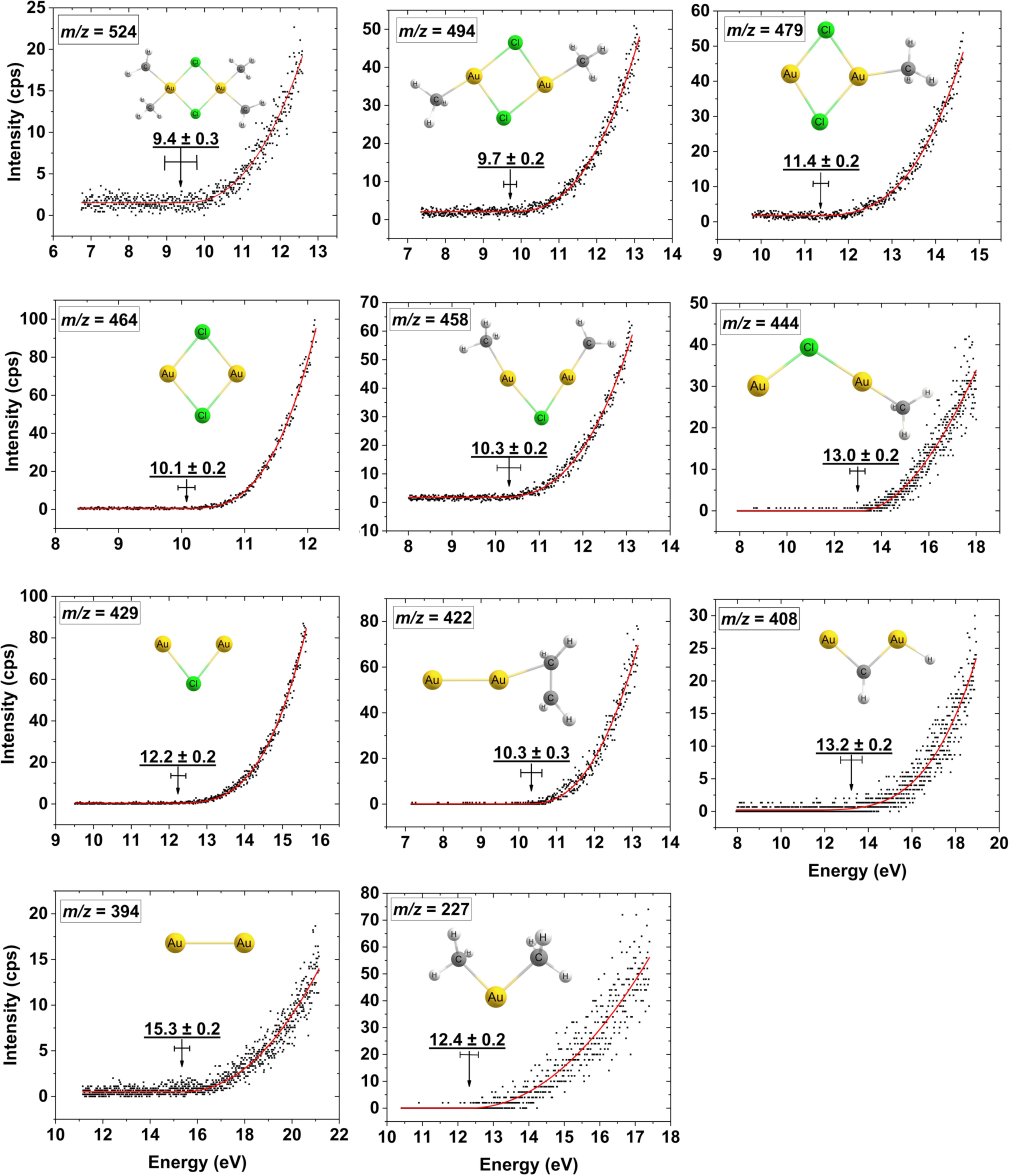
Representative fits to the onset region of the DI ion yield curves for the parent cation and the most dominant positively charged fragments from [Au(CH_3_)_2_Cl]_2_. The appearance energies and their confidence limits for each ion are shown along with the respective chemical structure optimized at the PBE0-TZVP level of theory.

For most of the observed *m*/*z* ratios, the assignment of the underlying fragmentation process is not straightforward as the neutral fragments, complementary to the *m*/*z* ratios observed, may be assigned to more than one composition. Thus, to better understand the underlying fragmentation process, the respective appearance energies (AEs) are determined using a Wannier-type threshold function (see the Methods section) and compared to calculated threshold energies for a variety of potential reaction pathways. A full list of all optimized geometries (Cartesian coordinates) of the parent and positively charged ions at the PBE0-TZVP level of theory are provided in Supporting Information File 1, Table S1. Figure 7 shows the fitted onset region of representative ion yield curves for the individual fragments along with their average AEs determined from fits to 3–4 ion yield curves recorded on different days. Also shown are the respective confidence limits and the structures of the respective positive ions optimized at the PBE0-TZVP level of theory.

Table 2 compares the individual AEs with calculated thresholds for different potential reactions leading to the respective fragments. Here the values for single bond ruptures without new bond formations are shown along with the best matches of the AEs with the respective threshold values. For comparison, threshold values for selected processes that are next to the assigned processes are also shown in Table 2. A complete set of all calculated threshold values, and the respective processes are shown in Supporting Information File 1, Table S2. The thresholds are calculated at both the PBE0-TZVP and DLPNO-CCSD(T)-TZVP levels of theory, as discussed in the Methods section. The assigned fragmentation reactions shown in Table 2 are in bold. In the assignment we primarily compare to the DLPNO-CCSD(T)-TZVP values. We note that as activation barriers may shift the AEs to higher values as compared to the respective thermochemical thresholds, the true thermochemical threshold may in some cases be lower than the respective AE. Where the current comparison does not allow the assignment to one combination of neutral fragments, the closest matches are in bold in Table 2.

**Table 2:** Comparison of the experimental AE values with the respective calculated threshold values. The threshold energies are calculated for homolytic bond ruptures and where no new bonds are formed the neutral fragments are the radical species.

*m*/*z*	Products	AE (eV)	PBE0-TZVP (eV)	DLPNO-CCSD(T)-TZVP (eV)

**524**	**[Au(CH** **_3_** **)** **_2_** **Cl]** **_2_** **^+^**	**9.4 ± 0.3**	**9.23**	**9.92**
**494**	[Au_2_Cl_2_(CH_3_)_2_]^+^ + 2CH_3_ **[Au_2_Cl_2_(CH_3_)_2_]^+^** **+ CH_3_CH_3_** [Au_2_Cl_2_(CH_3_)_2_]^+^ + CH_2_CH_2_ + H_2_	**9.7 ± 0.2**	13.51 **9.67** 11.28	14.06 **10.29** 11.65
**479**	[Au_2_Cl_2_(CH_3_)]^+^ + 3CH_3_ **[Au_2_Cl_2_(CH_3_)]^+^** **+ CH_3_** **+ CH_3_CH_3_** [Au_2_Cl_2_(CH_3_)]^+^ + CH_2_CH_2_ + H_2_ + CH_3_	**11.4 ± 0.2**	15.01 **11.18** 12.78	15.04 **11.27** 12.64
**464**	[Au_2_Cl_2_]^+^ + 4CH_3_ **[Au_2_Cl_2_]^+^** **+ 2CH_3_CH_3_** [Au_2_Cl_2_]^+^ + CH_3_CH_3_ + 2CH_3_ [Au_2_Cl_2_]^+^ + CH_2_CH_2_ + H_2_ + CH_3_CH_3_	**10.1 ± 0.2**	17.32 **9.65** 13.48 11.25	17.65 **10.12** 13.89 11.49
**458**	[Au_2_Cl(CH_2_CH_3_)]^+^ + 2CH_3_ + Cl + H **[Au_2_Cl(CH_2_CH_3_)]^+^** **+ CH_3_CH_3_** **+ HCl** **[Au_2_Cl(CH_2_CH_3_)]^+^** **+ CH_4_** **+ CH_3_Cl** [Au_2_Cl(CH_2_CH_3_)]^+^ + CH_3_CH_3_ + Cl + H	**10.3 ± 0.2**	18.72 **10.46** **10.64** 14.88	18.50 **10.41** **10.60** 14.73
**444**	[Au_2_Cl(CH_3_)]^+^ + Cl + 3CH_3_ **[Au_2_Cl(CH_3_)]^+^** **+ Cl + CH_3_CH_3_** **+ CH_3_** **[Au_2_Cl(CH_3_)]^+^** **+ CH_3_Cl + 2CH_3_** [Au_2_Cl(CH_3_)]^+^ + CH_3_Cl + CH_3_CH_3_	**13.0 ± 0.2**	17.42 **13.58** **13.72** 9.88	17.29 **13.52** **13.76** 9.99
**429**	[Au_2_Cl]^+^ + 4CH_3_ + Cl **[Au_2_Cl]^+^** **+ HCl + 2CH_4_** **+ CHCH_2_** **[Au_2_Cl]^+^** **+ CH_3_** **+ CH_3_Cl + CH_3_CH_3_** [Au_2_Cl]^+^ + 2CH_3_CH_3_ + Cl	**12.2 ± 0.2**	19.11 **12.54** **11.57** 11.44	18.84 **12.33** **11.54** 11.30
**422**	[Au_2_(CH_2_CH_2_)]^+^ + 2CH_3_ + 2Cl + 2H **[Au_2_(CH_2_CH_2_)]^+^** **+ 2ClCH_3_** **+ H_2_** [Au_2_(CH_2_CH_2_)]^+^ + CH_3_CH_3_ + 2HCl	**10.3 ± 0.3**	22.20 **10.53** 9.51	21.84 10.46 9.44
**408**	[Au(CH)AuH]^+^ + 3CH_3_ + 2Cl + H **[Au(CH)AuH]^+^** **+ CH_2_CH_2_** **+ 2HCl + CH_4_** **[Au(CH)AuH]^+^** **+ CH_3_CH_3_** **+ HCl + ClCH_3_**	**13.2 ± 0.2**	24.57 **13.35** **12.60**	24.14 **13.06** **12.54**
**394**	[Au_2_]^+^ + 4CH_3_ + 2Cl **[Au_2_]^+^** **+ 2CH_3_** **+ 2CH_3_Cl** **[Au_2_]^+^** **+ CH_2_CH_2_** **+ 2CH_3_** **+ 2HCl** **[Au_2_]^+^** **+ 2CH_3_CH_3_** **+ 2Cl** [Au_2_]^+^ + 2CH_3_CH_3_ + Cl_2_ [Au_2_]^+^ + CH_3_CH_3_ + 2CH_3_Cl	**15.3 ± 0.2**	22.39 **14.99** **15.56** **14.71** 11.99 11.15	22.09 **15.05** **15.39** **14.56** 12.14 11.28
**227**	[(CH_3_)Au(CH_3_)]^+^ + 2CH_3_ + 2Cl + Au **[(CH_3_)Au(CH_3_)]^+^** **+ CH_3_CH_3_** **+ Cl_2_** **+ Au** **[(CH_3_)Au(CH_3_)]^+^** **+ AuCl + CH_3_CH_3_** **+ Cl** [(CH_3_)Au(CH_3_)]^+^ + 2CH_3_Cl + Au [(CH_3_)Au(CH_3_)]^+^ + CH_2_CH_2_ + 2HCl + Au	**12.4 ± 0.2**	17.81 **11.24** **12.46** 10.41 10.98	18.72 **12.54** **12.20** 11.68 12.01

For the appearance energy of the parent cation [Au(CH_3_)_2_Cl]_2_^+^, (ionization energy of [Au(CH_3_)_2_Cl]_2_) we determine a value of 9.4 ± 0.3 eV. Within the confidence limit, this agrees well with the calculated threshold of 9.23 eV found at the PBE0-TZVP level of theory. However, at the DLPNO-CCSD(T)-TZVP levels of theory, we calculate a threshold of 9.92 eV, which is ≈0.2 eV above the upper confidence limit for the experimental AE.

For the loss of one methyl group, *m*/*z* 509, we find the intensity too low to determine the AE. However, for the loss of two methyl groups, *m*/*z* 494, we find an AE of 9.7 ± 0.3 eV. Considering only single bond ruptures (i.e., the formation of two CH_3_ radicals in this process) it results in threshold values of 13.51 and 14.06 eV at the PBE0-TZVP and DLPNO-CCSD(T)-TZVP levels of theory, respectively. These are ≈4 eV above the AE which is significantly higher than the confidence limits of the experiment.

However, considering the formation of ethane CH_3_CH_3_ in this process, it results in threshold values of 9.67 and 10.29 eV at the respective levels of theory. Similar to the parent ion, the density functional theory (DFT) value agrees well with the experimental AE, while the DLPNO-CCSD(T)-TZVP value is ≈0.3 eV above its higher confidence limit. We also calculated the threshold values at the DLPNO-CCSD(T) level using the smaller split valence polarization (SVP) basis set, and these values are given in Supporting Information File 1, Table S2. At that level, the agreement with the experimental ionization energy and the AE for the loss of two methyl groups is good. However, while the AEs for the more complex processes are generally well reproduced at the TZVP level, we found these to be underestimated when using the SVP basis set.

For the loss of three methyl ligands, *m*/*z* 479, we derive an AE of 11.4 ± 0.2, while the calculated threshold for this process without new bond formation is found to be 15.01 and 15.04 eV at the respectively levels of theory. Hence, also here new bonds must be formed for this process to be thermochemically possible at its AE.

Considering the formation of ethane from two of the methyl radicals, the threshold values are lowered to 11.18 and 11.27 eV, respectively, which agree well with the experimental AE at both levels of theory. For the final reaction in this progression (i.e., the loss of all four methyl ligands, *m*/*z* 464) we determine an AE of 10.1 ± 0.2 eV, while the threshold values without the consideration of new bond formations are approx. 7 eV higher at both levels of theory. Considering the formation of two ethane molecules in this process lowers these threshold values to 9.65 and 10.12 eV. Here the value at the DFT level of theory is somewhat below the confidence limit of the AE. However, the value at the DLPNO-CCSD(T)-TZVP level of theory agrees well with the experimental AE value.

The next progression observed in the mass spectrum, *m*/*z* 458, 444, and 429, constitutes a progressive loss of the methyl ligands along with the loss of one chlorine and partly additional hydrogen loss. We find the AEs for these channels to be 10.3 ± 0.2, 13.0 ± 0.2, and 12.2 ± 0.2 eV, respectively. Considering only single bond ruptures and no new bond formations results also here in threshold values that are considerably higher than the respective AEs. We have considered several potential reaction paths leading to these fragments and for *m*/*z* 458 (AE = 10.3 ± 0.2 eV), the formation of ethane and HCl, where the threshold values are 10.46 and 10.41 eV at the PBE0-TZVP and DLPNO-CCSD(T)-TZVP levels of theory, respectively, is in good agreement with the experimental AE. The formation of methane and chloromethane is the next closest match with thresholds of 10.64 and 10.60 eV at the respective levels of theory. Similarly, for the loss of chlorine and three methyl groups, *m*/*z* 444 (13.0 ± 0.2 eV), we get the closest agreements when considering the formation of ethane, atomic chlorine, and the methyl radical where the threshold values are 13.58 and 13.52 eV at the respective levels of theory. The formation of chloromethane and two methyl radicals, where the respective threshold values are 13.72 and 13.76 eV, are also considered. On the other hand, considering the formation of chloromethane and ethane brings the respective thresholds down to 9.88 and 9.99 eV, respectively. This is at both levels of theory approx. 3 eV below the experimental AE. Finally, for the formation of [Au_2_Cl]^+^, *m*/*z* 429 (12.2 ± 0.2 eV), we find the closest match with the experimental AE when considering significant rearrangements leading to the neutral counterparts HCl, 2CH_4_, and C_2_H_3_. The threshold value for this reaction is 12.54 and 12.33 eV at the PBE0-TZVP and DLPNO-CCSD(T)-TZVP levels of theory, respectively. However, this reaction requires, in addition to new bond formations, the migration of three hydrogens between the respective ligands lost. The respective threshold values for the formation of ethane, chloromethane, and the methyl radical as the neutral counterparts are 11.57 and 11.54 eV (i.e., 0.63 and 0.66 eV below the experimental AE) respectively. However, as discussed above, such extensive rearrangement reactions are likely to be associated with non-negligible activation barriers and may also be subject to kinetic shift of the AEs [46–48], making them appear at higher energies. Thus, we also consider the formation of ethane and chloromethane to be a potential reaction path for the formation of [Au_2_Cl]^+^. Considering the formation of two ethane molecules and the chlorine radical as the neutral counterparts, on the other hand, lowers these threshold values further, to 11.44 and 11.30 eV, respectively (i.e., approx. 1 eV below the experimental AE).

The next progression is that of the loss of both chlorine ligands and 2, 3, and 4 methyl ligands, and is partly associated with additional loss of hydrogen. The resulting positive ion fragments appear at *m*/*z* 422, 408, and 394, and are assigned to [Au_2_(C_2_H_4_)]^+^, [Au_2_(CH_2_)]^+^, and [Au_2_]^+^, respectively. The AE for *m*/*z* 422, [Au_2_(C_2_H_4_)]^+^, is found to be 10.3 ± 0.3 eV, which agrees with the threshold values of 10.53 and 10.46 eV calculated at the respective levels of theory when assuming the formation of two chloromethanes and one hydrogen molecule as the neutral counterparts. The threshold for the formation of ethane and two HCl molecules as the neutral counterparts is found to be 9.51 and 9.44 eV, respectively (i.e., 0.79 and 0.86 eV below the experimental AE). The *m*/*z* 408 constitutes the formation of [Au_2_(CH_2_)]^+^; that is, the loss of three methyl groups and one hydrogen. We derive an AE of 13.2 ± 0.2 eV for this fragment, which agrees well with the threshold values of 13.35 and 13.06 eV, calculated at the DFT and coupled cluster level of theory (TZVP), respectively, for the formation of ethene (CH_2_CH_2_), 2HCl, and methane as the neutral counterparts. Considering the formation of ethane (CH_3_CH_3_), HCl, and chloromethane as the neutral counterparts lowers the respective threshold values 12.60 to 12.54 eV (i.e., 0.40 and 0.46 eV below the lower confidence limit) respectively. Under the same considerations of potential activation barriers on these reaction paths, we do not exclude this reaction as a potential route for the formation of [Au_2_(CH_2_)]^+^. Finally, for the formation of [Au_2_]^+^, *m*/*z* 394, we derive an AE of 15.3 ± 0.2 eV. Similar to the formation of [Au_2_(CH_2_)]^+^, considering ethene (CH_2_CH_2_), two HCl, and two methyl radicals as the neutral counter parts, results in threshold values of 15.56 and 15.39 eV, at the respective levels of theory. From these, the coupled cluster value agrees well with the experimental AE, and the DFT value is only marginally above its higher confidence limit. Considering the formation of chloromethane and two methyl radicals gives threshold values of 14.99 and 15.05 eV, respectively. This also agrees with the AE at the coupled cluster level and is only slightly below the confidence limits at the DFT level. The formation of two ethanes and two atomic chlorines, on the other hand, gives values of 14.56 and 14.71 eV, respectively, which is approx. 0.5 and 0.4 eV below the lower limit of the AE for [Au_2_(CH_2_)]^+^. The formation of two ethanes and Cl_2_ or ethane and two chloromethanes as the neutral counterparts brings the threshold values approx. 3 eV below the experimental AE at both levels of theory. The last methyl loss progression constitutes the loss of one of the gold atoms, both chlorine and two, three, and four methyl ligands appearing in the mass spectrum at *m*/*z* 227/225, 212, and 197, respectively. From these, *m*/*z* 225 is attributed to the additional loss of two hydrogens as compared to *m*/*z* 227, and 197 is attributed to Au^+^. The intensities of these ion signals are comparatively low and the number of combinations of neutral fragments is large. Nonetheless, we have determined the AEs for *m*/*z* 227 and 225 (see Supporting Information File 1 for *m*/*z* 225). For *m*/*z* 227, we found the AE to be 12.4 ± 0.2 which agrees at the coupled cluster level of theory with the formation of ethane, Cl_2_, and atomic chlorine (12.54 eV) and with the formation of ethane, AuCl, and atomic chlorine (12.20 eV). At the coupled cluster level, however, the thresholds for the formation of ethene, 2 HCl, and atomic gold and for the formation of two chloromethanes and atomic gold are only 0.2 and 0.5 eV below the lower confidence limit of the experimental AE, respectively.

Several other possible combinations of neutral fragments were considered for all *m*/*z* ratios and a complete list of these can be found in Supporting Information File 1, Table S2.

It is clear from these considerations that the DI of [Au(CH_3_)_2_Cl]_2_ is dominated by rearrangement reactions with multiple bond ruptures and new bond formations. For the loss of the methyl groups without loss of chlorine, the assignment of the neutral counterparts is straightforward and is dominated by ethane formation from the respective methyl groups. For the additional loss of one or two chlorines, which is also in part associated with hydrogen loss, the assignment of the neutral counterparts is more complex. This is especially true as activation barriers are likely to influence the experimentally determined AEs of the respective cationic fragments, and considering the extent of these processes, kinetic shift may also play a role. Thus, reactions where the calculated thresholds are somewhat lower than the respective AEs cannot be excluded, and in many cases, we cannot offer a conclusive assignment to one single set of neutral counterparts. Notwithstanding, it is clear that the formation of ethane and hydrochloric acid and/or chloromethane plays an important role in these fragmentation processes.

**Figure 8:**
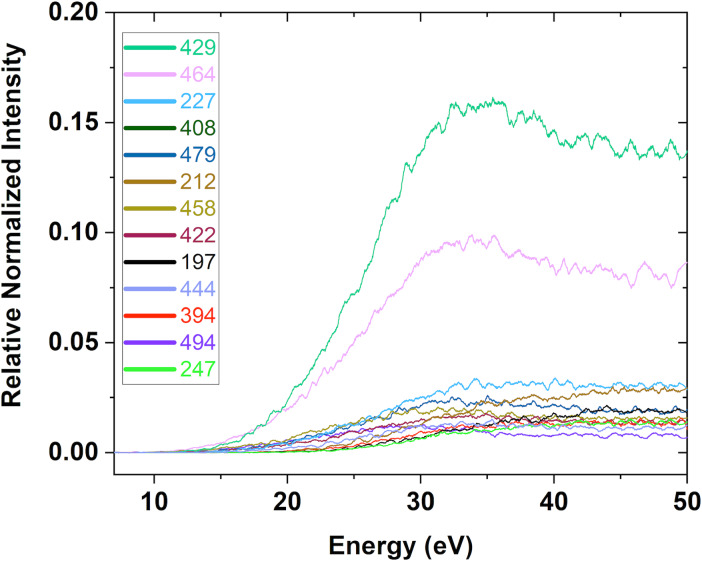
Positive ion yields for the most significant DI fragments from [Au(CH_3_)_2_Cl]_2_ in the incident electron energy range from below their threshold up to 50 eV. The ion yields are normalized with respect to the pressure and the signal intensity of Ar^+^ from Ar at 50 eV.

In FEBID, the effective damage yield [28,49] for a specific precursor will be a convolution of the energy distribution of the electrons involved (i.e., of the primary, secondary, and inelastic scattered electrons) and the energy dependence of the cross sections for the respective electron-induced processes. For more quantitative comparison with the UHV FEBID experiments presented here and the earlier HV experiments on this precursor, Figure 8 shows the energy dependence of the relative cross sections for the most significant DI processes from below their thresholds to 50 eV. The intensities are normalized to the pressure and the signal intensity of Ar^+^ from Ar at an electron energy of 50 eV.

Table 3 shows the integral intensities of these fragments over the presented energy range, along with their relative peak intensities as observed in the mass spectrum shown in Figure 6. These are normalized with respect to the highest intensity fragment *m*/*z* 429, [Au_2_Cl]^+^ (set as 100). At the bottom of the table, the relative intensities are translated to average carbon and chlorine loss by summing the contributions from all fragments weighted by their respective carbon and chlorine losses and dividing by the total intensity of all DI fragments. Finally, the expected average elemental composition of a deposit that would form only if these DI fragmentation processes were operational is shown. This is calculated from the relative intensities and composition of the gold containing positive ions and the neutral fragments, while desorption of all other fragments is assumed in this thought experiment. From the integrated intensity in the ion yield curves, we derive an average chlorine loss of 0.96 and an average carbon loss of 3.42, and from the mass spectra, these values are 0.92 and 3.39, respectively. This marginal difference reflects the lower contribution of the higher threshold fragments to the integral intensities as well as the shape of the respective ion yield curves.

**Table 3:** Relative intensities of DI fragments from [Au(CH_3_)_2_Cl]_2_ calculated from the peak intensities at 50 eV as they appear in the mass spectrum (Figure 6) and from the areas under the respective ion yield curves shown in Figure 7. The intensities are normalized with respect to the highest intensity fragment, *m*/*z* [Au_2_Cl]^+^, that is set as 100. Also shown is the composition of a hypothetical deposit that would be formed if the process would be governed by DI as observed in the gas phase. For comparison, the composition of the FEBID deposits from the current UHV and the previous HV experiments are shown at the bottom of the table.

*m*/*z*	Fragment	Relative DI Yield (Integration)	Relative DI Yield (intensity)	

494	[Au_2_Cl_2_(CH_3_)_2_]^+^	7.20	9.2	
479	[Au_2_Cl_2_(CH_3_)]^+^	15.13	19.02	
464	[Au_2_Cl_2_]^+^	62.36	67.08	
458	[Au_2_Cl(C_2_H_5_)]^+^	12.46	20.45	
444	[Au_2_Cl(CH_3_)]^+^	8.24	9.41	
429	[Au_2_Cl]^+^	100	100	
422	[Au_2_(C_2_H_4_)]^+^	10.78	11.45	
408	[Au_2_(CH_2_)]^+^	15.69	17.18	
394	[Au_2_]^+^	7.62	11.04	
247	[(CH_3_)AuCl]^+^	6.58	7.57	
227	[(CH_3_)_2_Au]^+^	19.97	17.8	
212	[(CH_3_)Au]^+^	13.65	9.82	
197	[Au]^+^	8.49	6.95	

Chlorine loss		0.96	0.92	
Carbon loss		3.42	3.39	

Expect. comp. DI	55 atom % Au	29 atom % Cl	16 atom % C	
UHV-FEBID	45–61 atom % Au	1–2 atom % Cl	38–49 atom % C	5–8 atom % Sn
HV-FEBID	29–41 atom % Au	2–6 atom % Cl	53–68 atom % C	

In the hypothetic deposition experiment, this would lead to a deposit composed of approx. 55 atom % Au, 29 atom % Cl, and 16 atom % C. With respect to the high gold content, this is in line with the UHV FEBID deposits. However, the carbon content is significantly lower than that observed in FEBID, and most noticeably, in both the UHV and HV depositions, the removal of chlorine is close to quantitative, while on average only half of the chlorine is cleaved from a parent molecule in DI. It is thus clear that the unaltered DI processes, as they are observed in the gas phase under single collision conditions, cannot explain the deposit composition observed in FEBID.

**Figure 9:**
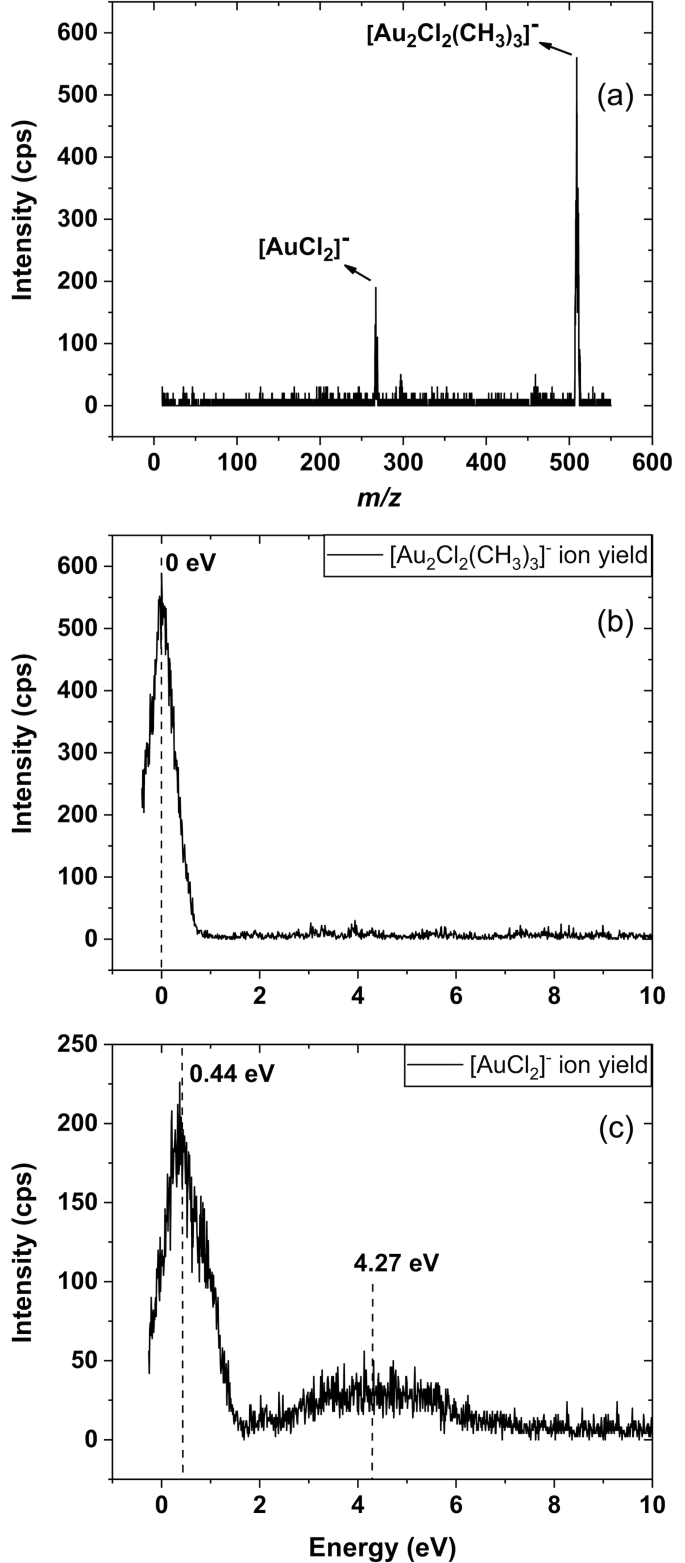
(a) Cumulative negative ion mass spectrum composed of the sum of individual mass spectra recorded at 1 eV intervals in the energy range from 0 to 8 eV and covering the *m*/*z* range from approx. 10 to 550. (b) and (c) Negative ion yield curves of the most significant fragments observed in DEA to [Au(CH_3_)_2_Cl]_2_ in the energy range from approx. 0 to 10 eV. (b) [Au_2_Cl_2_(CH_3_)_3_]^−^, *m*/*z* 509 and (c) AuCl_2_^−^, *m*/*z* 267.

To explore the potential role of DEA in the deposition formation, Figure 9a shows a negative ion mass spectrum in the *m*/*z* range from 10 to 550. As DEA is a resonant process, it proceeds within distinct energy ranges. Thus, to cover the relevant energy, the mass spectrum shown in Figure 9a is the sum of individual mass spectra recorded in the electron energy range from approx. 0 to 10 eV at 1 eV intervals. Figure 9b and Figure 9c show the respective ion yield curves for the dominating fragments in this energy range.

It is clear from Figure 9 that fragmentation through DEA is significantly less extensive than that observed in DI. In fact, only two significant channels are observed: the loss of a single methyl group leading to the formation of [Au_2_Cl_2_(CH_3_)_3_]^−^, *m*/*z* 509, which appears through a comparatively narrow contribution peaking at the 0 eV threshold, and the formation of AuCl_2_^−^, *m*/*z* 267, whose low energy contribution peaks at about 0.4 eV. In addition, a broader and lower intensity contribution to the AuCl_2_^−^ signal is observed at approx. 4 eV. In DEA the cross section for the attachment process is inversely proportional to the square root of the incident electron energy [25], and the attachment cross sections are highest at the 0 eV threshold. Consequently, exothermic DEA processes that proceed at the 0 eV threshold are generally the most efficient. This is also the case here, and we find the single methyl loss leading to the formation of [Au_2_Cl_2_(CH_3_)_3_]^−^ to be exothermic by 0.96 and 0.88 eV at the PBE0-TZVP and DLPNO-CCSD(T)-TZVP levels of theory, respectively. Similarly, we find the formation of [AuCl_2_]^−^ to be exothermic by approx. 2 eV at both levels of theory when presuming the formation of two neutral ethane molecules and elemental gold in the process. We also calculated the thresholds for the formation of Au(CH_3_)_2_ and one ethane molecule as well as AuCH_3_, ethane, and methane as the neutral counterparts in this process and also found these channels to be exothermic at both levels of theory. The calculated thresholds for the negative ion formation are shown in Supporting Information File 1, Table S2. From the width of the [Au_2_Cl_2_(CH_3_)_3_]^−^ and AuCl_2_^−^ contributions in their ion yield curves, we anticipate that these are from overlapping resonances associated with single electron occupation of the lowest lying unoccupied molecular orbitals. While the presumably faster loss of a single methyl group dominates at the threshold value, AuCl_2_^−^ is only produced through the high-energy flank. Applying the same considerations here as for the positive ions, we can calculate an expected elemental composition for a deposit that would be formed only if the unaltered DEA channels, as they proceed in the gas phase under single collision conditions, were active. By using the integrated intensities from the ion yield curves shown in Figure 9, and presuming that the elemental gold stays on the surface, the expected composition would be approx. 32 atom % gold, 32 atom % chlorine, and 36 atom % carbon. Hence, in DEA no chlorine loss is observed as compared to close-to-quantitative chlorine loss in the FEBID experiments, under HV as well as UHV.

### Gas phase, UHV and HV FEBID

When comparing FEBID deposits produced from [Au(CH_3_)_2_Cl]_2_ under UHV and HV conditions, it is apparent that the compositions are qualitatively the same (i.e., high gold content, close-to-quantitative removal of chlorine, and predominantly carbon residues). In the UHV experiments, however, the residual carbon is lower, as expected. Although not as markedly, the chlorine removal is also more complete under UHV. Despite the fact that both UHV and HV deposits qualitatively offer the same picture, it is clear that significantly higher gold content is achievable under UHV. It is also apparent that the composition of the deposit is dependent on the deposition current and the cleanliness of the substrate. Although we have not considered neutral dissociation upon electron excitation in the current gas-phase experiments, it is clear that the electron-induced fragmentation of [Au(CH_3_)_2_Cl]_2_ is strongly influenced in the FEBID experiments as compared to the single collision conditions in the gas phase. Interestingly, a more extensive fragmentation is observed in the FEBID experiment, while by considering only energy dissipation, one would rather expect stabilization (i.e., the opposite effect). In DI, rearrangement reactions are found to be dominant among the fragmentation processes, rather than direct dissociation without new bond formation. Such rearrangement reactions generally proceed along convoluted paths on the respective potential energy surfaces, which are likely to be altered in the condensed phase or on a substrate surface. Furthermore, considering the current electron dose of approx. 5 × 10^19^ *e*^−^/cm^2^ and the volume of the resulting deposits, electron-induced secondary reactions may also play a role. Assuming dense pacing and a molecular diameter of 1 nm, a deposit with an area of 4 × 4 μm^2^ and a height of 20 nm, consists of approx. 3 × 10^8^ molecules. The number of electrons that this volume has been exposed to is approx. 8 × 10^12^. Respectively, a monolayer consists of 1.5 × 10^7^ molecules that has been exposed to 4 × 10^11^ electrons. This corresponds to approx. 35,000 electrons per molecule. Assuming a generic cross section of 10^−16^ cm^2^, which is on the order of magnitude for DI and DEA of the FEBID precursor Co(CO)_3_(NO) [49–50] and Pt(PF_3_)_4_ [51], the reactive area of this monolayer is 0.15 μm^2^. Statistically, this implies approx. 300 reactive incidents per molecule if the cross section is assumed to stay unchanged. This is clearly not a quantitative assessment but shows that multiple electron collisions may play a role in FEBID, while the gas-phase experiments are conducted under single collision conditions. In the last decade, interest in organometallic FEBID precursors containing higher amounts of halogens, chlorine, bromine, and iodine has increased, and studies on the gold(I) precursors AuXL (L = P(NMe_2_)_3_, PMe_3_, CNMe, CN*t-*Bu, P(OCH_2_CF_3_)_3_; X = Cl, Br, I) [52], and on Ru(CO)_4_I_2_ [31], Ru(η^3^-C_3_H_5_)(CO)_3_X (X = Cl, Br) [48,53–54], Pt(NH_3_)_2_Cl_2_ [55–56], and Pt(CO)_2_X_2_ (X = Cl, Br) [57–61] have been reported. These include post-deposition purification studies [62–63], thin layer exposure to electrons at approx. 500 eV [64–65], gas-phase studies under single collision condition [28,66], and FEBID under HV and UHV conditions [30,60]. Most noticeably, platinum precursors Pt(CO)_2_X_2_ (X = Cl, Br) have been studied with respect to FEBID and their low-energy electron interaction under a variety of different conditions. Those are the gas phase [59,61] in thin layers under non-steady state conditions [57], in comparative FEBID experiments under HV and UHV conditions [60], and with respect to post-deposition purification through electron exposure and through reductive halogen removal using atomic hydrogen [58]. In an early study by Spencer et al. [57], 0.7 nm layers of Pt(CO)_2_Cl_2_ were exposed to 500 eV electrons and desorbing ligands were monitored by mass spectrometry, while the development of the deposit was monitored using XPS. It was found that the initial decomposition, up to an electron dose of approx. 10^16^ *e*^−^/cm^2^ was characterized by a rapid CO loss, leaving a deposit of approx. 1:2 Pt/Cl ratio. However, a prolonged exposure up to doses around 10^19^ *e*^−^/cm^2^, which is the order of magnitude applied here, led to an almost complete removal of the halogen. While the first step was in good agreement with the observations in the gas phase under single collision conditions, the second step indicated that pure deposits were achievable through prolonged electron exposure of the deposit formed in the first step. This was further explored in a post-deposition purification study where two approaches were taken: prolonged electron exposure and reductive halogen removal using atomic hydrogen [58]. While atomic hydrogen was found to effectively remove the halogen, prolonged electron exposure was only found to have significant influence at the surface of the deposits. The halogen content in the bulk, on the other hand, was not markedly reduced. Interestingly, a recent comparative deposition study using SEM under HV and an Auger spectrometer under UHV showed significant differences in the composition of the deposits [60]. While the deposits fabricated in the UHV chamber primarily contained halogen contamination and comparatively low carbon content, carbon was the main component in the deposits under HV, while the halogen content was as low as 7.5–8 atom %. It was pointed out that this might be due to a reductive removal of the halogen through reactions with background water, which is of considerably higher concentration in the HV chamber than in the UHV chamber. While HCl formation is apparently significant in DI of [Au(CH_3_)_2_Cl]_2_ in the current experiment, and may in part be responsible for the etching effects observed, there are no indications that the higher background water content in the HV experiments influences the composition of the deposits. Electron-induced secondary reactions, on the other hand, may play a role, especially as the growth rate of the deposits are comparatively low, and correspondingly the electron exposure of each monolayer is high. This could be probed in a non-steady-state experiment similar to those reported for Pt(CO)_2_X_2_ (X = Cl, Br) and several other potential FEBID precursors [57].

## Conclusion

In the current study, we evaluated the performance of [Au(CH_3_)_2_Cl]_2_ as a precursor for gold deposition in FEBID in an UHV setup at different beam currents, but at a constant dose and on different substrates. The elemental composition of the deposits was determined by AES and their morphology and crystal structure was examined using SEM, AFM, HAADF-STEM, and SAED. Complementary DI and DEA experiments were carried out under single collision conditions in the gas phase to better understand the underlying electron-induced processes, quantum chemical threshold calculations were used to aid the interpretation of the gas-phase data.

Generally, we find [Au(CH_3_)_2_Cl]_2_ suitable for FEBID with respect to both its volatility and stability, and no indication of decomposition is observed in the DI experiments. Interestingly, a complete chlorine removal is observed in the FEBID experiments, and a gold content as high as 61 atom % Au was obtained with a beam current of 1.5 nA on a Si(111) substrate precleaned by Ar^+^ sputtering. On an untreated SiO_2_(500 nm)/Si(111) surface at 0.4, 1.5, and 3 nA, the gold content of the deposits was found to be 45, 50, and 52 atom % Au, respectively. In a previous FEBID study under HV conditions [33], these were found to be 29 and 41 atom % Au at beam currents of 0.1 and 0.4 nA, respectively. Here, a close-to-quantitative removal of chlorine was also observed. Thus, it is clear that the gold content is dependent on the cleanliness of the substrate, on the deposition environment in general, and on the electron beam current. With respect to the morphology, the deposits were found to be composed of a bimodal size distribution of predominantly spherical nanoparticles with the dominant component being uniformly distributed particles of less than 5 nm diameter, and a nonuniform distribution of larger particles (10–15 nm). The deposit was found to be polycrystalline, with the close-packed face-cantered cubic crystal structure of bulk gold. In the gas phase we found DEA to be at large limited to single methyl loss leading to the observation of [M – CH_3_]^−^, and the formation of AuCl_2_^−^. Hence, no chlorine loss was observed in DEA. On the other hand, dissociative ionization was found to be extensive and governed by rearrangement reactions and new bond formations. These are largely associated with neutral ethane formation. Formation of HCl is also likely to be significant, although definite distinction between channels where HCl was formed and where CH_3_Cl was formed was not always provided. On average, only 50% of the chlorine was lost from the central gold atoms in DI and none in DEA, as compared to the close-to-quantitative chlorine loss in FEBID. Contrarily, more efficient methyl loss was observed in DI than was reflected in the FEBID deposits. We note that neutral dissociation upon electron excitation was not probed here. Nonetheless, it is clear that chlorine loss through electron-induced fragmentation in the FEBID experiments is considerably more extensive than what may be accounted for under single collision conditions in the gas phase. We anticipate that this is due to electron-induced secondary and tertiary reactions, and that the initial step in the fragmentation process is rather dominated by DI than by DEA. This is a hypothesis that can readily be probed in non-steady state experiments, similar to those conducted by Spencer et al. [57] for Pt(CO)_2_Cl_2_. In such experiments, electron-dose dependence of the composition of thin layers of *cis-*[Au(CH_3_)_2_Cl]_2_ can be monitored by means of XPS, and neutral desorbing fragments may be detected by means of mass spectrometry. This would also allow definite distinction between fragmentation channels where HCl is formed and where CH_3_Cl is formed. The latter is an important parameter due to potential etching effects through HCl formation, and generally as HCl outgassing may also cause instrumental problems. Furthermore, with the current synthesis and purification protocol, a carryover of tin, whose origin we attribute to the use of Sn(CH_3_)_4_ as a methylation agent, was observed in the deposits. These impurities could not be identified (but are likely chlorinated Sn compounds, such as SnCl_4_ or SnCl_2_) and were not fully removed through recrystallization or drying under vacuum. This may influence the morphology of the deposits. For future FEBID studies or applications with [Au(CH_3_)_2_Cl]_2_, the current synthesis and purification protocols need to be refined. For example, by using different methylating agents, such as MeMgCl or Me_3_Bi, or by using an established indirect route in which Au(CH_3_)_2_OH is acidified with an HCl solution [67].

Overall, [Au(CH_3_)_2_Cl]_2_ is found to have good potential for the fabrication of high gold content deposits in FEBID. However, as observed for other FEBID precursors, aliphatic ligands are generally not good leaving groups. Thus, to reduce the carbon content of depositions from [Au(CH_3_)_2_Cl]_2_, in situ or post-deposition purification protocols would need to be incorporated into the deposition process. Furthermore, it would be advantageous to better understand the mechanism of halogen removal as that might be critical with respect to the achievable growth rates.

## Methods

### Precursor synthesis and precursor handling

[Au(CH_3_)_2_Cl]_2_ was synthesized by slightly modifying the procedure described by Paul and Schmidbaur [36]. The synthesis was performed under a nitrogen atmosphere using standard Schlenk techniques, methanol (>99%), and predried pentane. The starting material H[AuCl_4_]·3H_2_O was obtained in the form of orange crystals by dissolving gold metal in aqua regia, evaporating all liquids and, after the addition of concentrated HCl, evaporating all liquids again.

Orange crystals of H[AuCl_4_]·3H_2_O (1.97 g, 5.00 mmol) were dissolved in approx. 25 mL of methanol and the solution was cooled to −80 °C. A solution of SnMe_4_ (2.81 g, 16.26 mmol) in approx. 25 mL of methanol was precooled to −80 °C and rapidly added to the stirred solution of H[AuCl_4_]·3H_2_O. The reaction mixture was slowly warmed to −50 °C and kept at this temperature for 20 h while stirring. During the reaction, the temperature was monitored with an electronic thermometer directly in the reaction mixture (not in the cooling bath). Subsequently, 3.0 mL of concentrated HCl in approx. 20 mL of methanol was slowly added, while keeping the temperature below −40 °C. Then, the reaction mixture was slowly allowed to warm up to room temperature over approx. 2 h. After removal of all volatiles in vacuum at room temperature, an off-white product with some metallic gold particles was isolated. The raw product was extracted three times with 20 mL portions of dry pentane. The pentane extract was isolated by filtration, and after removal, the product [Au(CH_3_)_2_Cl]_2_ was obtained as an off-white powder. The obtained yield of dimeric [Au(CH_3_)_2_Cl]_2_ was: 0.51 g, 0.97 mmol, 39%. The slightly dark color is likely due to contamination with colloidal Sn^0^ and/or Au^0^, which could not be removed by filtration or crystallization. [Au(CH_3_)_2_Cl]_2_ (*M*_w_ 524.98): calcd for C_4_H_12_Au_2_Cl_2_: C, 9.15; H, 2.30; found: C, 8.93; H, 2.21; ^1^H NMR (400 MHz, C_6_D_6_, 25 °C) δ = 1.02 ppm (s); mp 72 °C (lit. 73 °C) [36].

The so synthesized [Me_2_AuCl]_2_ precursor was kept at 253 K and filled into a stainless-steel precursor reservoir under nitrogen atmosphere (glove box). The reservoir with a small glass-window was chosen to visually check the precursor quality and possible degradation. The filled reservoir was directly attached to the analysis chamber and wrapped in aluminum foil to avoid photodecomposition during the experiments.

### Deposition and characterization

The FEBID structures were fabricated in a commercial UHV system (Multiscanlab, Omicron Nanotechnology, Germany) with a base pressure of *p* < 2 × 10^−10^ mbar. The system consists of a UHV compatible electron column (Leo Gemini) for SEM (nominal resolution higher than 3 nm), electron-beam-based lithography (EBL, FEBID), and local AES using a hemispherical electron energy analyzer with a resolution higher than 10 nm. The Auger spectra (magnification: 100.000×; spectra area: 1.2 × 0.9 μm^2^) were recorded with an acceleration voltage of 15 kV and a beam current of 3 nA. Electron exposures for FEBID were performed at a beam energy of 5 keV and beam currents of 0.4, 1.5, and 3 nA. A quadrupole mass spectrometer (Pfeiffer / Prisma QMS 200M) is integrated in the system and was used to acquire mass spectra (MS) of the gas-phase [Au(CH_3_)_2_Cl]_2_ precursor at room temperature (298 K). The lithographic processes were controlled through a custom-developed software, based on LabVIEW 8.6 (National Instruments) and a high-speed DAC PCIe-card (M2i.6021-exp, Spectrum GmbH, Germany) [68]. The lithographic parameters were a step size of 6.2 nm and a sweep number of 10. The dwell times applied to the FEBID structures were calculated as electron doses and are reported in the Results and Discussion section. The SEM images were acquired at a beam energy of 15 keV and at a current of 0.4 nA with a SmartSEM (Zeiss). Minor contrast and brightness adjustments were applied.

The precursor gas was dosed onto the sample surface through a nozzle. During the FEBID process, the precursor container was held at room temperature as the precursor, [Au(CH_3_)_2_Cl]_2_, was found to be sufficiently volatile to be transferred from the container into the UHV chamber via the gas-injection system. For the experiments, the precursor pressure in the chamber was adjusted to 6.0–9.0 × 10^−7^ mbar. Based on simulations using the GIS Simulator (version 1.5) [69], the local pressure at the sample surface was estimated to be approx. 30 times that of the chamber pressure, resulting in a local pressure at the substrate surface of approx. 2.0–3.0 × 10^−5^ mbar.

The FEBID structures were deposited onto and investigated on two different substrates: SiO_2_ (500 nm)/Si(111) and thermally cleaned Si(111). These substrates are commonly used in FEBID and were chosen for a better comparison with previous experiments. Specifically, SiO_2_ was used for comparison with a previous FEBID study with [Au(CH_3_)_2_Cl]_2_ which was conducted under HV conditions on SiO_2_. No specific preparation was applied to clean the SiO_2_ (500 nm)/Si(111) sample. The Si(111) surface, on the other hand, was cleaned by 45 min Ar^+^ sputtering (*V*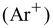 = 1 keV, *P*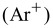 = 4 × 10^−6^ mbar) and annealed up to 823 K under an oxygen atmosphere for 90 min (HF etching was not applied). Atomic force microscopy was performed with a JPK NanoWizard 4 by using the noncontact mode, and an FEI Titan Themis³ 300 transmission electron microscope was used to obtain HAADF-STEM and SAED results.

### Gas-phase experiments

The gas-phase DI and DEA experiments were conducted under single collision conditions in a crossed electron/molecular beam instrument which has been described in detail elsewhere [70] and only a short description is given here. It consists of an effusive stainless-steel capillary gas inlet system, a trochoidal electron monochromator, and a quadrupole mass spectrometer (Hiden EPIC 1000) with a detection system, allowing for operation in positive or negative ion mode. The system is housed in a HV chamber with a typical base pressure of 2 × 10^−8^ mbar. In the interaction section of the chamber, the electron beam crosses the effusive molecular beam and charged products generated in the respective electron–molecule interactions are extracted into a quadrupole mass spectrometer which is orthogonal to both the molecular beam and the electron beam. The working pressure during the current experiments was maintained at approx. 4 × 10^−7^ mbar. The TEM was kept at 393 K with two halogen lamps to prevent condensation of precursor molecules or background contaminations on the components of the electrical lens. The electron energy resolution was around 140 meV, as determined from the full width at half maximum (FWHM) of the SF_6_^−^ formation from SF_6_ at a 0 eV incident energy. Mass spectra were recorded at fixed electron energies by scanning through the relevant *m*/*z* range, and ion yields were recorded at a fixed *m*/*z* ratios by scanning through the respective electron energy range. Positive ion MS were recorded at a 50 eV incident electron energy and not at 70 eV as is conventional in EI mass spectrometry. This was due to limitations posed by the computer-controlled energy ramp. However, as 50 eV is already well above the maximum cross section for all fragments formed, as can be seen from Figure 8, this does not influence the current results. Negative ion MSs were recorded at 0, 1, 2, … 10 eV incident electron energies. In negative ion mode the electron energy scale was calibrated by the SF_6_^−^ formation from SF_6_ at 0 eV, and in positive ion mode by the ionization energy of Ar [71]. The positive ion yields were normalized relative to the target gas pressure and the Ar^+^ signal intensity from Ar at 50 eV, and the negative ion yields to the target pressure and the signal intensity of SF_6_^−^ from SF_6_ at 0 eV. Appearance energies of positive ions were calculated by fitting the onset of the respective ion yields with a Wannier type function [72] of the form:




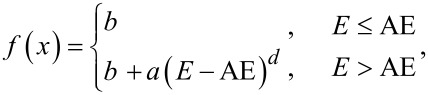




where AE denotes the appearance energy, *E* the incident electron energy, *b* counts for constant background signal, *a* is a scaling coefficient, and *d* is an exponential factor describing the onset region. The AE values reported are the average from fits to 3 ion yield curves recorded on different days and the standard deviation reported is that of the respective averages rounded up to the nearest 100 meV. The confidence limits reported are set by visual inspection to bracket the onset of the individual curves and are equal to or higher than the standard deviations from the fittings.

### Quantum chemical calculations

The ORCA program, version 4.1, was used as the platform for all quantum chemical computations [73]. For all geometry optimization we used the hybrid GGA functional PBE0 with the def2-TZVP basis set and the D3BJ dispersion correction developed by Grimme [74]. This was based on an evaluation of DFT functionals for gold(I) and gold(III) hydrocarbon by Kang et al. [75] who found the hybrid GGA functional PBE0 with a TZ basis set to be the best for geometry optimization. In a later study by Kepp et al. [76], it was found that PBE and TPSS functionals with dispersion corrections generally perform well for evaluation of gold bond dissociation enthalpies. The restricted Kohn–Sham (RKS) formalism was used for closed-shell systems and the unrestricted (UKS) for open-shell systems. Tight SCF settings were applied in the geometry optimizations and the TPSS/def2-TZVP single point energies were calculated with normal SCF settings. Positive harmonic vibrational frequencies derived at the PBE0/def2-TZVP level of theory demonstrated that all structures of the parent molecule and its fragments were stationary points on the respective potential energy surfaces. These frequencies were then used to compute zero-point vibrational energies and thermal corrections. For a variety of FEBID precursors, the optimization of the geometry at the DFT level of theory and the determination of the energy of the system using post-HF approaches, such as CCSD(T), have been reported earlier [46,48,61]. In the current study, single-point coupled-cluster computations were also done on the optimized geometries. These were done at the DLPNO-CCSD(T)/TZVP level of theory using the TZVP/c auxiliary basis set and were carried out with normal PNO settings.

Threshold energies for individual processes were calculated at both levels of theory by subtracting the single point energies of the optimized geometries of the respective fragments formed from those of the parent molecule, including the respective ZPVEs and thermal energy corrections in all cases. Several alternative reaction paths, including rearrangement reactions were calculated for all fragmentation processes considered.

## Supporting Information

Additional details on the preparation of lamella for STEM and SAED experiments (Supporting Information File 1, Figure S1). The particle size analysis by Feret diameter method (Supporting Information File 1, Figure S2). The comparison of particle numbers versus particle diameters with respect to the different beam currents (Supporting Information File 1, Figure S3). The line profiles of AFM image (Supporting Information File 1, Figure S4). The AES results on Si(111) substrate before and after surface treatment by Ar^+^ sputtering (Supporting Information File 1, Figure S5). The Cartesian coordinates (Å) of optimized geometries and their respective Gibbs free energy (Eh) calculated at the PBE0/def2-TZVP level of theory (Supporting Information File 1, Table S1). Full table of calculated threshold values for potential reaction pathways leading to the observed cations and anions due to DI and DEA processes at the PBE0/def2-TZVP, DLPNO-CCSD(T)-SVP, and DLPNO-CCSD(T)-TZVP levels of theory (Supporting Information File 1, Table S2).

File 1 Additional figures and tables.
