# Supplementary material for: A combined gas-phase dissociative ionization, dissociative electron attachment and deposition study on the potential FEBID precursor [Au(CH3)2Cl]2
Source: Beilstein J Nanotechnol. 2023 Dec 6;14:1178–99. doi: 10.3762/bjnano.14.98 (PMC10714508; doi:10.3762/bjnano.14.98)
Supplement: File 1 — Additional figures and tables. [file Beilstein_J_Nanotechnol-14-1178-s001.pdf]

BEILSTEIN JOURNAL OF NANOTECHNOLOGY

Supporting Information

for

A combined gas-phase dissociative ionization, dissociative electron attachment and deposition study on the potential FEBID precursor $[Au(CH_3)_2Cl]_2$

Elif Bilgilisoy, Ali Kamali, Thomas Xaver Gentner, Gerd Ballmann, Sjoerd Harder, Hans-Peter Steinrück, Hubertus Marbach and Oddur Ingólfsson

Beilstein J. Nanotechnol. **2023,** *14,* 1178–1199. doi:10.3762/bjnano.14.98

Additional figures and tables

License and Terms: This is a supporting information file under the terms of the Creative Commons Attribution License (https://creativecommons.org/licenses/by/4.0). Please note that the reuse, redistribution and reproduction in particular requires that the author(s) and source are credited and that individual graphics may be subject to special legal provisions.
The license is subject to the *Beilstein Journal of Nanotechnology* terms and conditions: (https://www.beilstein-journals.org/bjnano/terms)

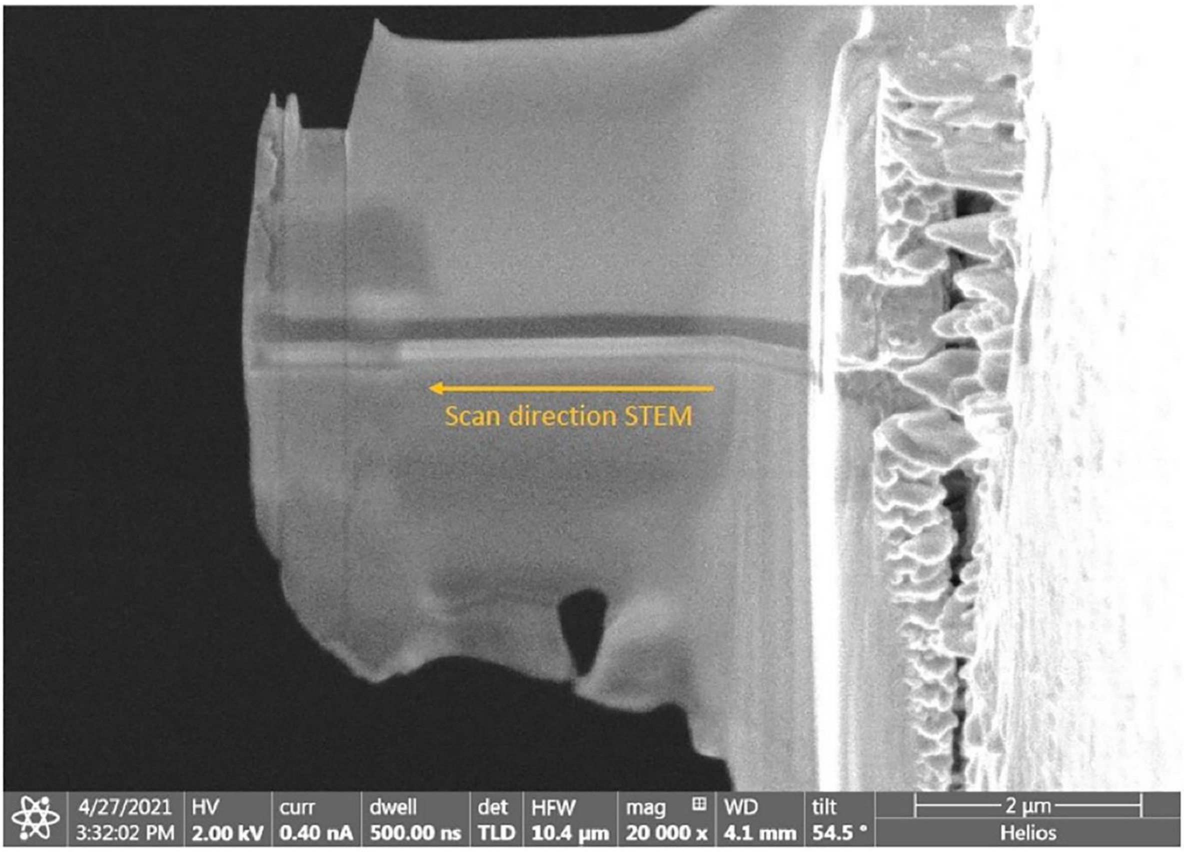

Figure S1: Prepared lamella for STEM and SAED experiments.

The observed gold nanoparticles (Figure 1d) have irregular shapes. Therefore, the diameter of the selected particle was defined by considering the perpendicular distance between two parallel tangent lines of the particle at an arbitrary angle. This particle size analysis method is known as Feret diameter or caliper diameter. Beside the diameter of the particles, their approximate amount in one SEM image was also acquired by firstly removing the background from the SEM image, then applying the image threshold, and finally counting the numbers automatically (Figure S2).

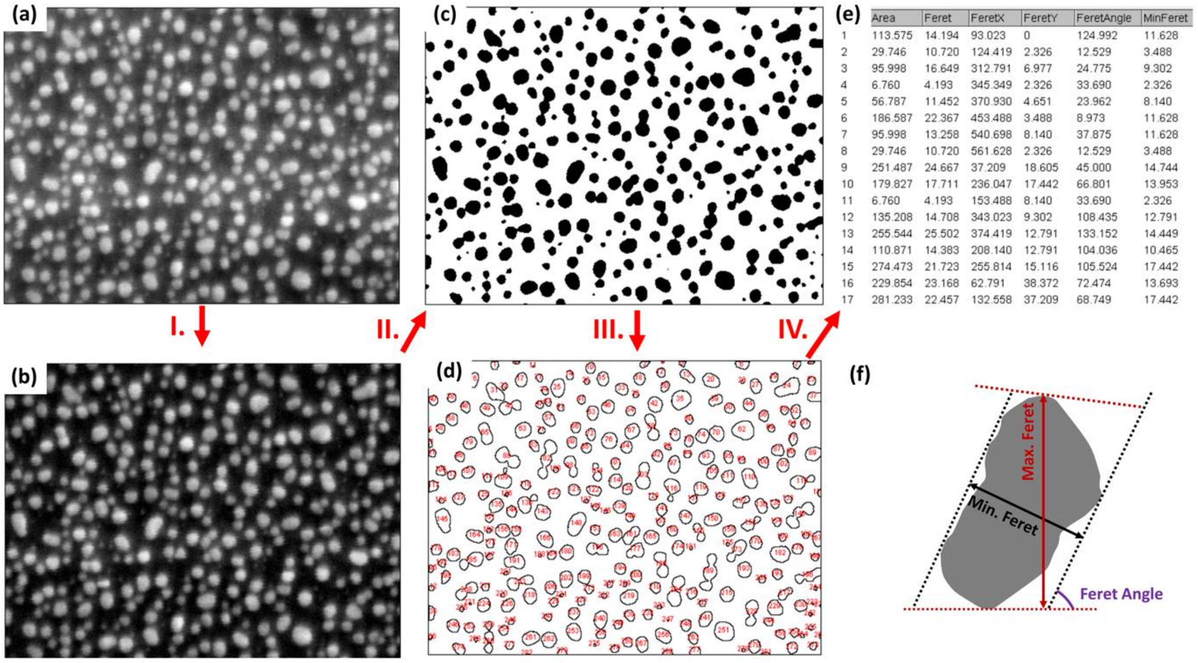

	Area	Feret	FeretX	FeretY	FeretAngle	MinFeret
1	113.575	14.194	93.023	0	124.992	11.628
2	29.746	10.720	124.419	2.326	12.529	3.488
3	95.998	16.649	312.791	6.977	24.775	9.302
4	6.760	4.193	345.349	2.326	33.690	2.326
5	56.787	11.452	370.930	4.651	23.962	8.140
6	186.587	22.367	453.488	3.488	8.973	11.628
7	95.998	13.258	540.698	8.140	37.875	11.628
8	29.746	10.720	561.628	2.326	12.529	3.488
9	251.487	24.667	37.209	18.605	45.000	14.744
10	179.827	17.711	236.047	17.442	66.801	13.953
11	6.760	4.193	153.488	8.140	33.690	2.326
12	135.208	14.708	343.023	9.302	108.435	12.791
13	255.544	25.502	374.419	12.791	133.152	14.449
14	110.871	14.383	208.140	12.791	104.036	10.465
15	274.473	21.723	255.814	15.116	105.524	17.442
16	229.854	23.168	62.791	38.372	72.474	13.693
17	281.233	22.457	132.558	37.209	68.749	17.442

Figure S2: (a) An SEM image of FEBID gold nanoparticles. (b) Background-subtracted SEM image of (a) using ImageJ. (c) Image after the automatic threshold was applied in ImageJ program. (d) and (e) Results of automatic particle count in the program. (f) Illustration of the Feret parameters: minimum Feret (min. Feret), maximum Feret (max. Feret), and Feret angle.

The comparative histogram graphs of particle numbers versus particle diameters with respect to the beam currents for 400 pA (blue bars), 1.5 nA (green bars) and 3 nA (purple bars) were obtained from SEM and AFM, and plotted in Figure S3.

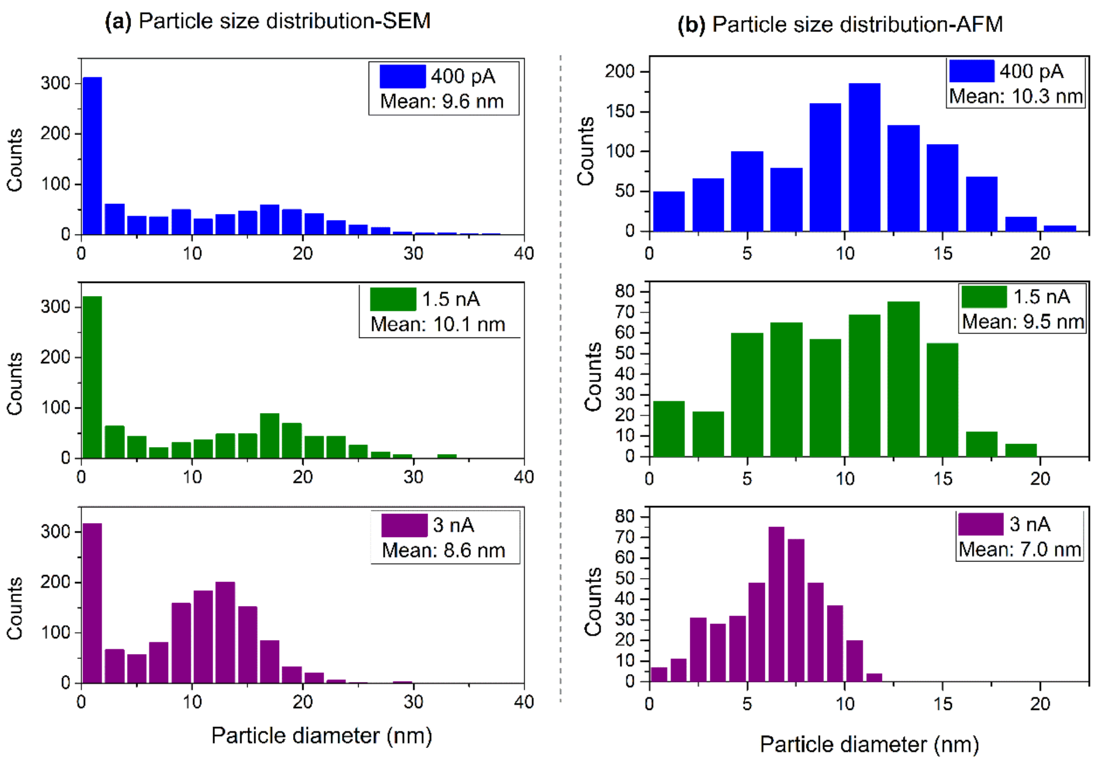

Figure S3: (a) and (b) The particle size distributions obtained from SEM and AFM, respectively, for the FEBID gold nanoparticles with respect to the different applied beam currents.

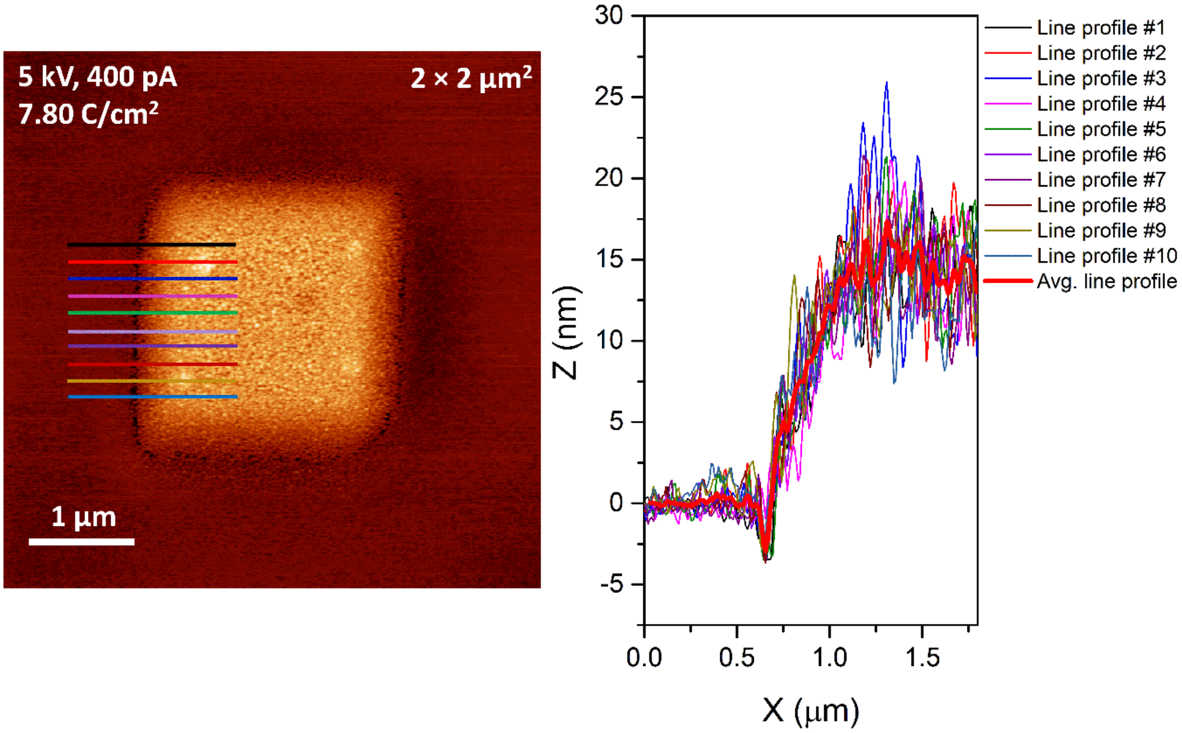

Figure S4: The AFM image of the FEBID deposit (5 kV, 400 pA) is shown on the left, and the line profiles from 10 different lines and their average line profile are shown on the right.

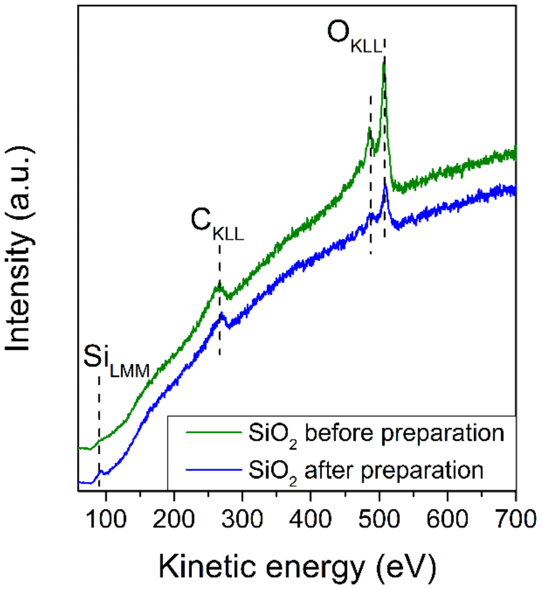

Figure S5: AES on Si(111) before surface treatment (green line) and after surface treatment (45 min. Ar^+ sputtering + 90 min. annealing at 823 K under oxygen) on the same substrate (blue line).

Table S1: All optimized geometries (parent molecule and cations) and their Gibbs free energy (Eh) at the PBE0/ def2-TZVP level of theory, expressed as Cartesian coordinates (Å).

Molecule	Cartesian coordinates (Å)				Final Gibbs free energy (Eh)
$[Au_2Cl_2(CH_3)_4]$	Au	-2.072104	3.052679	-0.000000	-1351.07736325
	Au	-1.989186	-0.547564	0.000000	
	Cl	-3.700537	1.214412	-0.000000	
	Cl	-0.360753	1.290702	-0.000000	
	C	-0.673402	4.528115	0.000000	
	H	-1.112834	5.522029	0.000000	
	H	-0.068504	4.373457	-0.893801	
	H	-0.068504	4.373457	0.893801	
	C	-3.481400	4.517269	0.000000	
	H	-4.446950	4.013642	0.000000	
	H	-3.365518	5.129161	-0.893266	
	H	-3.365518	5.129161	0.893266	
	C	-3.387887	-2.023002	0.000000	
	H	-2.948454	-3.016916	0.000008	
	H	-3.992779	-1.868350	-0.893805	
	H	-3.992789	-1.868340	0.893796	
	C	-0.579889	-2.012155	0.000000	
	H	0.385662	-1.508527	-0.000000	
	H	-0.695771	-2.624047	-0.893266	
	H	-0.695770	-2.624046	0.893267	
$[Au_2Cl_2(CH_3)_4]^+$	Au	-2.035627	2.919475	-0.027570	-1350.73933480
	Au	-2.017481	-0.444636	-0.010941	
	Cl	-3.788694	1.232543	0.016664	
	Cl	-0.261716	1.250851	-0.054282	
	C	-0.669548	4.439482	-0.345837	
	H	-1.190608	5.274419	-0.803414	
	H	0.084423	4.008933	-1.000436	
	H	-0.281488	4.671763	0.645097	
	C	-3.414131	4.432888	0.268665	
	H	-4.246110	3.972802	0.796011	
	H	-3.684834	4.768818	-0.731350	
	H	-2.929968	5.205118	0.858956	
	C	-3.388970	-1.973133	-0.245290	
	H	-2.926816	-2.738725	-0.861040	
	H	-4.248317	-1.518273	-0.731478	
	H	-3.615120	-2.321717	0.761195	
	C	-0.632326	-1.956939	0.242645	
	H	0.172322	-1.510011	0.821366	
	H	-0.322947	-2.225852	-0.766396	
	H	-1.114845	-2.775127	0.767926	
$[Au_2Cl_2(CH_3)_2]^+$	Au	-1.819984	2.875611	0.004036	-1271.03956804
	Au	-2.287517	-0.353586	0.107295	
	Cl	-3.785590	1.579411	0.201733	
	Cl	-0.320606	0.946395	-0.090320	
	C	-2.858006	4.635135	0.063053	
	H	-3.352792	4.697531	1.029611	
	H	-3.556767	4.643787	-0.770184	
	H	-2.043623	5.357541	-0.051498	
	C	-1.251219	-2.114009	0.047678	

<table>
<tr><td></td><td>H -0.553619 -2.123453 0.882026
H -0.754647 -2.175376 -0.918170
H -2.065026 -2.837038 0.159960</td><td></td></tr>
<tr><td>[Au₂Cl₂(CH₃)]⁺</td><td>Au -2.710731 3.628260 1.202848
Au -0.876681 0.582329 -0.199154
Cl -2.761916 -1.062272 -0.912648
Cl -1.072379 2.990585 -0.250808
C -1.119442 -1.403061 -0.364717
H -1.118450 -1.935881 0.580988
H -0.527731 -1.861705 -1.150811
H 0.579778 0.504658 0.312600</td><td>-1231.21077897</td></tr>
<tr><td>[Au₂Cl₂]⁺</td><td>Au -2.056269 2.768478 0.000000
Au -2.005021 -0.263367 0.000000
Cl -3.870060 1.221887 -0.000000
Cl -0.191230 1.283232 -0.000000</td><td>-1191.35489549</td></tr>
<tr><td>[Au₂Cl(CH₂CH₃)]⁺</td><td>Au -2.333092 3.153032 0.004622
Au -2.219375 -0.696612 0.077322
Cl -0.911089 1.215951 0.078406
C -3.533568 4.824477 -0.052434
H -3.801526 4.928425 -1.104208
H -4.372147 4.586128 0.601986
H -2.880646 5.612803 0.325635
C -3.225313 -2.301311 0.078777
H -2.770456 -3.294805 0.109287
H -4.316942 -2.343808 0.049542</td><td>-731.29110113</td></tr>
<tr><td>[Au₂Cl(CH₃)]⁺</td><td>Au -1.973262 2.076357 -0.883223
Au 1.414636 0.775184 0.287775
Cl -0.345118 2.376157 0.689014
C 2.984080 -0.502369 -0.097905
H 3.660789 0.071733 -0.729953
H 2.520563 -1.355607 -0.594045
H 3.369621 -0.715024 0.900903</td><td>-771.12783263</td></tr>
<tr><td>[Au₂Cl]⁺</td><td>Au -1.995645 3.106475 0.000000
Au -1.918943 -0.598947 0.000001
Cl -0.602069 1.281921 0.000001</td><td>-731.29110113 Eh</td></tr>
<tr><td>[Au₂(CH₂CH₂)]⁺</td><td>Au -4.755924 0.426633 2.421132
Au -2.189804 -0.011332 1.982566
C -6.841047 1.019418 2.105349
C -6.728617 0.480247 3.374092
H -6.990154 -0.556436 3.562073
H -6.617365 1.122366 4.242251
H -7.193350 0.415805 1.274756
H -6.820579 2.094239 1.955491</td><td>-349.72187310</td></tr>
<tr><td>[Au(CH)AuH]⁺</td><td>Au -4.297039 1.251104 -0.422872
C -2.616996 0.259825 -0.471263
Au -1.544376 1.861455 -0.360334
H -2.365271 -0.655309 0.065992
H -0.076828 1.338935 -0.313894</td><td>-310.36432548</td></tr>
<tr><td>[Au₂]⁺</td><td>Au -0.191712 -0.032106 1.367387</td><td>-271.50548729</td></tr>
</table>

	Au -0.191712 -0.032106 -1.267660	
$[(CH_3)Au(CH_3)]^+$	Au -4.797108 0.324695 0.130111<br>C -3.255948 1.646757 0.222182<br>H -2.412115 0.993246 -0.015651<br>H -3.236292 2.025981 1.240925<br>H -3.425177 2.410875 -0.531572<br>C -6.214769 1.781065 0.177246<br>H -7.104844 1.195111 0.421352<br>H -6.236158 2.210645 -0.821382<br>H -5.947248 2.489221 0.956170	-215.12577260
$[AuCl_2]^-$	Au -1.936113 2.153547 0.000000<br>Cl -4.222798 2.061043 0.000000<br>Cl 0.350565 2.246094 0.000000	-1056.07074733
$[Au_2Cl_2(CH_3)_3]^-$	Au -1.294616 3.653728 -1.120733<br>Au -1.721580 -0.240883 -0.919247<br>Cl -1.767896 1.626343 -2.419054<br>Cl 0.273929 4.438163 -2.736831<br>C -0.978834 5.308899 0.018036<br>H -1.893686 5.902582 0.034097<br>H -0.166180 5.863241 -0.446048<br>H -0.720249 5.003606 1.032234<br>C -2.641690 3.056969 0.300580<br>H -3.530829 2.701840 -0.221069<br>H -2.897767 3.863223 0.987765<br>H -2.197334 2.222438 0.845948<br>C -1.675251 -1.832857 0.344300<br>H -0.649151 -2.187884 0.486364<br>H -2.271075 -2.664314 -0.046302<br>H -2.078061 -1.558642 1.325165	-1311.34276020

Table S2: Full list of experimental DI and DEA threshold values compared to theoretically calculated threshold values at PBE0/def2-TZVP, DLPNO-CCSD(T)/TZVP, and DLPNO-CCSD(T)/SVP levels of theory for the $[(CH_3)_2AuCl]_2$.

m/z	Products	AE (eV)	PBE0-TZVP (eV)	DLPNO-CCSD(T)-SVP (eV)	DLPNO-CCSD(T)-TZVP (eV)
**Dissociative Ionization**					
524	**$[Au_2Cl_2(CH_3)_4]^+$**	**9.4 ± 0.3**	**9.23**	**9.69**	**9.92**
494	$[Au_2Cl_2(CH_3)_2]^+ + 2(CH_3)$	**9.7 ± 0.2**	13.51	13.69	14.06
	**$[Au_2Cl_2(CH_3)_2]^+ + CH_3CH_3$**		**9.67**	**9.86**	**10.29**
	$[Au_2Cl_2(CH_3)_2]^+ + CH_2CH_2 + H_2$		11.28	11.36	11.65
479	$[Au_2Cl_2(CH_3)]^+ + 3(CH_3)$	**11.4 ± 0.2**	15.01	14.59	15.04
	**$[Au_2Cl_2(CH_3)]^+ + (CH_3) + CH_3CH_3$**		**11.18**	**10.76**	**11.27**
	$[Au_2Cl_2(CH_3)]^+ + CH_2CH_2 + H_2 + (CH_3)$		12.78	12.26	12.64
464	$[Au_2Cl_2]^+ + 4(CH_3)$	**10.1 ± 0.2**	17.32	17.06	17.65
	**$[Au_2Cl_2]^+ + 2(CH_3CH_3)$**		**9.65**	**9.41**	**10.12**
	$[Au_2Cl_2]^+ + CH_3CH_3 + 2(CH_3)$		13.48	13.23	13.89
	$[Au_2Cl_2]^+ + CH_2CH_2 + H_2 + CH_3CH_3$		11.25	10.91	11.49
458	$[Au_2Cl(CH_2CH_3)]^+ + 2CH_3 + Cl + H$	**10.3 ± 0.2**	18.72	17.87	18.50
	$[Au_2Cl(CH_2CH_3)]^+ + CH_3CH_3 + Cl + H$		14.88	14.04	14.73
	**$[Au_2Cl(CH_2CH_3)]^+ + CH_3CH_3 + HCl$**		**10.46**	**9.88**	**10.41**
	**$[Au_2Cl(CH_2CH_3)]^+ + (CH_4) + CH_3Cl$**		**10.64**	**10.23**	**10.60**
	$[Au_2Cl(CH_2CH_3)]^+ + 2(CH_3) + HCl$		15.46	16.01	15.41
	$[Au_2Cl(CH_2CH_3)]^+ + (CH_3) + CH_3Cl + H$		15.02	14.58	14.97
444	$[Au_2Cl(CH_3)]^+ + Cl + 3(CH_3)$	**13.0 ± 0.2**	17.42	16.52	17.29
	**$[Au_2Cl(CH_3)]^+ + Cl + CH_3CH_3 + (CH_3)$**		**13.58**	**12.69**	**13.52**
	**$[Au_2Cl(CH_3)]^+ + Cl(CH_3) + 2(CH_3)$**		**13.72**	**13.23**	**13.76**
	$[Au_2Cl(CH_3)]^+ + Cl(CH_3) + CH_3CH_3$		9.88	9.40	9.99
429	$[Au_2Cl]^+ + 4(CH_3) + Cl$	**12.2 ± 0.2**	19.11	18.00	18.84
	$[Au_2Cl]^+ + (CH_3) + CH_3Cl + CH_3CH_3$		11.57	10.89	11.54
	**$[Au_2Cl]^+ + HCl + 2(CH_4) + CHCH_2$**		**12.54**	**11.60**	**12.33**
	$[Au_2Cl]^+ + 2\ CH_3CH_3 + Cl$		11.44	10.35	11.30
	$[Au_2Cl]^+ + Cl + CH_3CH_3 + 2(CH_3)$		15.27	14.18	15.07
	$[Au_2Cl]^+ + 3(CH_3) + CH_3Cl$		15.41	14.72	15.31

422	$[Au_2(CH_2CH_2)]^+ + 2(CH_3) + 2Cl + 2H$	**10.3 ± 0.3**	22.20	20.87	21.84
	**$[Au_2(CH_2CH_2)]^+ + 2Cl(CH_3) + H_2$**		**10.53**	**10.09**	**10.46**
	$[Au_2(CH_2CH_2)]^+ + CH_3CH_3 + 2HCl$		9.51	8.72	9.44
	$[Au_2(CH_2CH_2)]^+ + 2CH_3 + 2HCl$		13.34	12.55	13.21
	$[Au_2(CH_2CH_2)]^+ + 2(CH_3) + Cl_2 + H_2$		15.21	14.75	15.10
	$[Au_2(CH_2CH_2)]^+ + CH_3CH_3 + Cl_2 + H_2$		11.37	10.93	11.33
408	$[Au_2(CH_2)]^+ + 3(CH_3) + 2Cl + H$	**13.2 ± 0.2**	24.57	23.42	24.14
	**$[Au_2(CH_2)]^+ + CH_2CH_2 + 2HCl + CH_4$**		**13.35**	**12.62**	**13.06**
	$[Au_2(CH_2)]^+ + 3(CH_3) + HCl + Cl$		20.14	19.26	19.83
	$[Au_2(CH_2)]^+ + CH_3CH_3 + CH_3 + HCl + Cl$		16.30	15.43	16.06
	$[Au_2(CH_2)]^+ + CH_3CH_3 + HCl + Cl(CH_3)$		12.60	12.15	12.54
	$[Au_2(CH_2)]^+ + 2(CH_3) + HCl + Cl(CH_3)$		16.44	15.97	16.30
	$[Au_2(CH_2)]^+ + CH_2CH_2 + HCl + Cl(CH_3) + H_2$		14.20	13.64	13.90
	$[Au_2(CH_2)]^+ + CH_3 + 2Cl(CH_3) + H$		17.16	16.84	17.10
407	$[Au_2(CH)]^+ + 3(CH_3) + 2H + 2Cl$	**13.8 ± 0.2**	28.82	27.63	28.40
	$[Au_2(CH)]^+ + 3(CH_3) + 2HCl$		19.96	19.32	19.76
	$[Au_2(CH)]^+ + CH_3CH_3 + CH_3 + 2HCl$		16.13	15.49	15.99
	$[Au_2(CH)]^+ + (CH_3) + 2Cl(CH_3) + H_2$		17.15	15.97	16.13
	$[Au_2(CH)]^+ + CH_3CH_3 + Cl + Cl(CH_3) + H_2$		17.02	16.32	16.78
	$[Au_2(CH)]^+ + CH_2CH_2 + Cl + Cl(CH_3) + 2H_2$		18.62	17.82	18.14
	**$[Au_2(CH)]^+ + CH_3CH_3 + Cl^- + Cl(CH_3) + H_2$**		**13.75**	14.46	13.69
	$[Au_2(CH)]^+ + 2HCl + CH_2CH_3 + CH_4$		15.92	15.35	15.85
394	$[Au_2]^+ + 4(CH_3) + 2Cl$	**15.3 ± 0.2**	22.39	21.94	22.09
	**$[Au_2]^+ + 2[CH_3CH_3] + 2Cl$**		**14.71**	**14.28**	**14.56**
	**$[Au_2]^+ + 2(CH_3) + 2(CH_3Cl)$**		**14.99**	**15.36**	**15.05**
	**$[Au_2]^+ + CH_2CH_2 + 2(CH_3) + 2HCl$**		**15.56**	**15.48**	**15.39**
	$[Au_2]^+ + 4(CH_3) + Cl_2$		19.66	20.02	19.68
	$[Au_2]^+ + 2[CH_3CH_3] + Cl_2$		11.99	12.36	12.14
	$[Au_2]^+ + CH_3CH_3 + 2(CH_3Cl)$		11.15	11.53	11.28
	$[Au_2]^+ + CH_2CH_2 + H_2 + 2(CH_3Cl)$		12.75	13.03	12.65
	$[Au_2]^+ + CH_2CH_2 + CH_3CH_3 + 2HCl$		11.72	11.66	11.62
227	$[(CH_3)Au(CH_3)]^+ + 2(CH_3) + 2Cl + Au$	**12.4 ± 0.2**	17.81	17.90	18.72
	**$[(CH_3)Au(CH_3)]^+ + CH_3CH_3 + Cl_2 + Au$**		**11.24**	**12.15**	**12.54**
	**$[(CH_3)Au(CH_3)]^+ + AuCl + CH_3CH_3 + Cl$**		**12.46**	**11.63**	**12.20**
	$[(CH_3)Au(CH_3)]^+ + 2(CH_3) + Cl_2 + Au$		15.08	15.98	16.31

	$[(CH_3)Au(CH_3)]^+ + CH_3CH_3 + 2Cl + Au$		13.97	14.07	14.95
	$[(CH_3)Au(CH_3)]^+ + 2Cl(CH_3) + Au$		10.41	11.32	11.68
	$[(CH_3)Au(CH_3)]^+ + CH_2CH_2 + 2HCl + Au$		10.98	11.44	12.01
	$[(CH_3)Au(CH_3)]^+ + AuCl + HCl + CH_2CH_2 + H$		13.90	13.16	13.58
225	$[(CH_2)Au(CH_2)]^+ + AuCl + Cl + 2(CH_3) + H_2$	**12.6 ± 0.4**	16.45	15.52	16.04
	**$[(CH_2)Au(CH_2)]^+ + CH_3CH_3 + Au + Cl_2 + H_2$**		**12.80**	**12.22**	**12.61**
	**$[(CH_2)Au(CH_2)]^+ + CH_3CH_3 + AuCl + H + HCl$**		**12.44**	**11.73**	**12.28**
	**$[(CH_2)Au(CH_2)]^+ + CH_3CH_3 + AuCl + Cl + H_2$**		**12.61**	**11.69**	**12.27**
	$[(CH_2)Au(CH_2)]^+ + 2Cl(CH_3) + Au + H_2$		11.96	11.22	11.59
	$[(CH_2)Au(CH_2)]^+ + 2Cl(CH_3) + AuH + H$		13.39	12.76	13.24
**Dissociative Electron Attachment**					
m/z	Products	AE (eV)	PBE0-TZVP (eV)	DLPNO-CCSD(T)-SVP (eV)	DLPNO-CCSD(T)-TZVP (eV)
**267**	$[AuCl_2]^- + Au + 2CH_3CH_3$	0.1-0.45	-2.20	-2.24	-1.96
	$[AuCl_2]^- + Au(CH_3) + CH_3CH_3 + CH_3$		-0.80	-0.71	-0.67
	$[AuCl_2]^- + (CH_3)Au(CH_3) + CH_3CH_3$		-1.82	-1.39	-1.54
**509**	$[M – CH_3]^- + CH_3$	0-0.2	-0.96	-0.76	-0.88
